# On the Inadequacy of the Current Transgenic Animal Models of Alzheimer’s Disease: The Path Forward

**DOI:** 10.3390/ijms25052981

**Published:** 2024-03-04

**Authors:** Vladimir Volloch, Sophia Rits-Volloch

**Affiliations:** 1 Department of Developmental Biology, Harvard School of Dental Medicine, Boston, MA 02115, USA; 2 Division of Molecular Medicine, Children’s Hospital, Boston, MA 02115, USA; 3 Department of Biological Chemistry and Molecular Pharmacology, Harvard Medical School, Boston, MA 02115, USA

**Keywords:** Alzheimer’s disease (AD), conventional AD, unconventional AD, amyloid cascade hypothesis (ACH), ACH-based models of AD, ACH-based AD drugs, ACH2.0, ACH2.0-based models of AD, intraneuronal Aβ (*i*Aβ), Aβ protein precursor (AβPP), AβPP-independent *i*Aβ production pathway

## Abstract

For at least two reasons, the current transgenic animal models of Alzheimer’s disease (AD) appear to be patently inadequate. They may be useful in many respects, the AD models; however, they are not. First, they are incapable of developing the full spectrum of the AD pathology. Second, they respond spectacularly well to drugs that are completely ineffective in the treatment of symptomatic AD. These observations indicate that both the transgenic animal models and the drugs faithfully reflect the theory that guided the design and development of both, the amyloid cascade hypothesis (ACH), and that both are inadequate because their underlying theory is. This conclusion necessitated the formulation of a new, all-encompassing theory of conventional AD—the ACH2.0. The two principal attributes of the ACH2.0 are the following. One, in conventional AD, the agent that causes the disease and drives its pathology is the intraneuronal amyloid-β (*i*Aβ) produced in two distinctly different pathways. Two, following the commencement of AD, the bulk of Aβ is generated independently of Aβ protein precursor (AβPP) and is retained inside the neuron as *i*Aβ. Within the framework of the ACH2.0, AβPP-derived *i*Aβ accumulates physiologically in a lifelong process. It cannot reach levels required to support the progression of AD; it does, however, cause the disease. Indeed, conventional AD occurs if and when the levels of AβPP-derived *i*Aβ cross the critical threshold, elicit the neuronal integrated stress response (ISR), and trigger the activation of the AβPP-independent *i*Aβ generation pathway; the disease commences only when this pathway is operational. The *i*Aβ produced in this pathway reaches levels sufficient to drive the AD pathology; it also propagates its own production and thus sustains the activity of the pathway and perpetuates its operation. The present study analyzes the reason underlying the evident inadequacy of the current transgenic animal models of AD. It concludes that they model, in fact, not Alzheimer’s disease but rather the effects of the neuronal ISR sustained by AβPP-derived *i*Aβ, that this is due to the lack of the operational AβPP-independent *i*Aβ production pathway, and that this mechanism must be incorporated into any successful AD model faithfully emulating the disease. The study dissects the plausible molecular mechanisms of the AβPP-independent *i*Aβ production and the pathways leading to their activation, and introduces the concept of conventional versus unconventional Alzheimer’s disease. It also proposes the path forward, posits the principles of design of productive transgenic animal models of the disease, and describes the molecular details of their construction.

## 1. Theory of a Disease and the Theory-Guided Disease Models Are Inextricably Entangled: The Current Transgenic Animal AD Models Are Inadequate Because the Underlying Theory Is

The ultimate objective of this study is to introduce a new class of transgenic AD models. The design of this class of models is informed by the novel, recently proposed theory of conventional AD—the amyloid cascade hypothesis 2.0 (ACH2.0) [1,2,3,4,5,6]. The major aspects and attributes of the ACH2.0 are described below. The present section elaborates the reasons that necessitated the formulation of this new theory, which in turn defines the principles of design of the adequate model systems.

### 1.1. The Theory of AD Defines the Construction of the Model Systems and Determines the Design of Potential Drugs: These Three Aspects Are Inextricable

Any model system of a disease is based upon and reflects the theory of that disease. The criteria of its success are simple: the pathology exhibited by it must epitomize the phenomenology of the actual disease. Accordingly, the failure of a model to faithfully reproduce the disease is indicative of the inadequacy of the underlying theory. By the same reasoning, the efficiency of drugs in the model system is only the first test. Drugs are expected to be efficient in the model system since both are based on the same theory. The real test of a drug comes with its implementation in the actual disease. If, despite its effectiveness in the model system, it were inefficient in the disease, this also would be indicative of the inadequacy of the underlying theory. Below, we examine the properties of the transgenic animal AD models and of the AD drugs designed and developed within the framework of the amyloid cascade hypothesis (ACH) theory of the disease.

### 1.2. The Amyloid Cascade Hypothesis: AD Is Caused and Driven by Extracellular Aβ Produced Solely in the AβPP Proteolytic/Secretory Pathway

The ACH was proposed over thirty years ago, in 1992 [7]. Its authors, Hardy and Higgins, defined it as follows: “Our hypothesis is that deposition of amyloid-β protein, the main component of the plaques, is the causative agent of Alzheimer’s pathology and that the neurofibrillary tangles, cell loss, vascular damage, and dementia follow as the direct result of this deposition” [7]. When the ACH was proposed, the occurrence and composition of Aβ plaques had been known for a considerable time. The immediate principal basis for its formulation was the discovery of a mutation that affected the generation of Aβ in the AβPP proteolytic pathway [8]; this mutation segregated with and apparently caused AD [8]. The ACH theory of AD appeared, at the time of its formulation, to be consistent with the accumulated data; it served as the basis for the design of transgenic animal models of AD and the development of the candidate AD drugs.

### 1.3. The Current ACH-Based Transgenic AD Models Could Be Useful but Are Not Adequate

The ACH-guided approach to the design of the transgenic mouse models of AD was simple: express as much as possible of human Aβ, preferentially in the mouse neurons or CNS, and this will cause its overexpression and excessive secretion and extracellular deposition, and the disease will follow. This approach was, in fact, carried out. Numerous copies of DNA encoding human AβPP, under the control of a powerful CNS-specific promoter, were introduced into the mouse genome. As a result, Aβ was indeed overproduced and over-secreted. This caused the excessive deposition of extracellular Aβ plaques, but symptomatic manifestation of the disease was rather limited. Whereas certain neurodegeneration and cognitive impairments were observed, the formation of the neurofibrillary tau tangles, the major hallmark of AD, was not seen. In an attempt to address this issue, additional mutations known to cause the early onset of AD (familial AD, FAD) were introduced into the transgenes; those included not only AβPP/Aβ mutations but also mutations in the presenilins (PSEN) involved in the processing of AβPP. The effect, however, was only quantitative, such as earlier manifestation of neurodegeneration and of limited cognitive impairments; no NFT formation was observed in any animal model. Clearly, AD appears to entail more than the increased production/secretion of Aβ in the AβPP proteolytic pathway, even in combination with the FAD mutations. Yet, for reasons discussed below, Aβ is apparently both the cause and the driver of the disease. Moreover, as described in the following section, some cognitive defects observed in the current transgenic animal models of AD, such as impaired memory formation and learning are not necessarily AD-specific.

### 1.4. Elicitation of the Integrated Stress Response (ISR) in Neurons, Triggered by Aβ or Otherwise, Results in the Impairment of Synaptic Plasticity, Long-Term Memory Formation, and Learning

The integrated stress response (ISR) is an evolutionary conserved signaling pathway, which is activated in response to different environmental and pathological conditions [9,10,11,12,13,14,15,16,17,18]. Those include nutrient deprivation, variable stresses, such as oxidative stress, inflammation, viral infection, protein aggregation and misfolding, and protein homeostasis defects. The numerous and various stressors capable of the eliciting the ISR converge on a single event, namely, the phosphorylation of the eukaryotic initiation factor 2 alpha (eIF2α) at a specific position (serine residue 51), hence the “integrated” in the “integrated stress response”. The elicitation of the ISR has far-reaching consequences: It reprograms and transforms both the transcriptional and translational landscapes of the affected cell. The former includes the activation of several transcription factors, and the latter results in a drastic reduction in total cellular protein synthesis via suppression of its 5′ cap-assisted initiation and the activation of cap-independent translation of a small subset of mRNA species.

The consequences of the ISR-initiated cellular reprogramming are numerous and some, relevant to the present subject, are discussed in the following sections below. One of these consequences is the impairment of synaptic plasticity, long-term memory formation and learning, all processes requiring de novo protein synthesis (suppressed by the ISR), in the affected organisms. The ISR and the consequent impairments are universally associated with AD and transgenic AD models, as well as with a variety of cognitive disorders, including Parkinson’s disease, Huntington disease, amyotrophic lateral sclerosis (ALS), traumatic brain injury, Down syndrome, prion disease, and Charcot–Marie–Tooth disease [19,20,21,22,23,24,25,26,27,28,29,30,31,32,33,34,35]. A mutation in the eIF2α phosphatase causing its increased phosphorylation and consequently the ISR is associated with a severe cognitive impairment [36]. The accumulated evidence that the observed cognitive impairments are consequences of the ISR is exceptionally strong: the genetic or pharmacological inhibition of the ISR prevents them, and systemic suppression of the ISR with the small-molecule inhibitor ISRIB alleviates them in model systems including the current transgenic AD models [37,38,39,40,41,42,43,44,45]. As discussed below, in AD patients and in the current transgenic models of AD, the elicitation of the ISR in neurons and its cognitive repercussions is mediated by Aβ. Importantly, however, the elicitation of the ISR in neurons and the resulting cognitive impairment could be triggered by stressors other than Aβ, as seen, for example, in cases of traumatic brain injury.

### 1.5. ACH-Based AD Drugs Should Work in the ACH-Based AD Models and so They Did, Spectacularly

The ACH theory of AD suggested the design of drugs for the treatment of the condition, referred to henceforward as ACH-based drugs. The central presumption of the ACH is that sufficiently high levels of extracellular Aβ cause AD. Therefore, the rationale for the construction of ACH-based drugs is straightforward: reduce levels of extracellular Aβ and you will abrogate the disease. Since both models and drugs are based on the same theory, they could be expected to be compatible, and indeed they were. Numerous agents capable of such reduction have been generated over the time. The most effective of them fall into two categories. The first consists of various antibodies that sequester Aβ in different forms, both soluble and insoluble. The second category of ACH-based drugs includes agents that suppress the production of Aβ in the AβPP proteolytic/secretory pathway. The less Aβ is generated, the less is secreted, and in conjunction with the physiologically occurring extracellular Aβ clearance, its levels would be reduced. Many of the candidate ACH-based AD drugs were spectacularly effective in the current transgenic AD models [46,47,48]. They not only stopped the symptomatic progression of the disease, but in some cases also reversed it. These results engendered the hope that a successful therapy for the disease could be at hand.

### 1.6. ACH-Based Drugs Failed Utterly in the Treatment of Symptomatic AD

However, all ACH-based drugs failed in human clinical trials of symptomatic AD [49,50] as spectacularly as they succeeded on the transgenic models (the effects of two apparent exceptions, lecanemab and donanemab, are consistent with the above statement and are discussed below and elsewhere [3,4,5,6]). Importantly, these drugs failed not because they could not do in human AD patients what they did in animal models. To the contrary, in both situations (AD models and AD patients), ACH-based drugs fulfilled their mechanistic mission very effectively, reducing substantially, up to 80%, levels of extracellular Aβ in AD patients [49,50]. The fact that the drugs were utterly ineffective in human AD patients reinforces the conclusion formulated above, namely, that the current transgenic models do not recapitulate AD and that the disease occurs significantly differently from its portrayal in the ACH theory. Taken cumulatively, the above reasoning suggests that neither current transgenic models of AD nor ACH-based AD drugs are adequate because their foundation, the ACH theory of the disease, is not.

### 1.7. Extracellular Aβ Can Be Ruled Out as the Causative Agent of AD

The results of clinical trials of ACH-based drugs, i.e., their complete inefficiency in treatment of symptomatic AD, indicate that extracellular Aβ is not the causative agent of AD. Indeed, the removal of the bulk of extracellular Aβ resulting in no therapeutic benefit can hardly be interpreted in any other way. This conclusion is strongly supported by the observation that there is, in fact, no good correlation between the levels of extracellular Aβ and the occurrence of the disease. Indeed, in a substantial fraction of the general population, up to 40%, extracellular Aβ accumulates, physiologically and in an aging-dependent manner, to the levels equal to or exceeding those seen in AD without causing any cognitive or neurodegenerative issues [51,52,53,54,55,56,57]. Moreover, the reverse is also correct, as follows from the observation of AD cases lacking the excessive accumulation of extracellular Aβ [58]. Taken cumulatively, these observations indicate emphatically that extracellular Aβ can be ruled out as the causative and driving agent of AD.

## 2. Amyloid Cascade Hypothesis 2.0

### 2.1. Intraneuronal Aβ Causes and Drives AD

In over thirty years since the discovery of the first Aβ-associated AD (FAD)-causing mutation [8], many more AD-causing mutations (and one that protects from the disease [59,60]) were detected. They occur either within Aβ or within AβPP in the vicinity of its Aβ segment, or within the presenilins. The common feature of these mutations is that they all, without exception, affect either the production or the structure of Aβ. With a single exception, they all cause the early onset of AD. The only exception is the Icelandic mutation. It changes a single amino acid residue in Aβ and this change is sufficient to confer to the carriers of this mutation protection from AD [59,60]. These observations leave very little doubt regarding the centrality and the causative role of Aβ in the disease. Since, as discussed above, extracellular Aβ can be ruled out as the causative agent of AD, this role falls to another pool of Aβ, the physiologically occurring intraneuronal Aβ—*i*Aβ. This inference is, in fact, supported by numerous studies indicating that intraneuronal *i*Aβ, rather than its extracellular counterpart, correlates with and is the major component of AD [61,62,63,64,65,66,67,68,69,70,71,72,73].

### 2.2. The Principal Attributes of the ACH2.0 Theory of AD

The rationale for and the strongest indication of the principal attributes of this novel theory of AD can be derived from the results of clinical trials of ACH-based drugs in general and from those of verubecestat in particular. Indeed, in AD patients, a substantial reduction in the levels of extracellular Aβ had no efficacy whatsoever. It follows that extracellular Aβ is neither the cause nor the driver of AD. Since, as discussed above, AD appears nevertheless to both cause and drive the disease, it has to be the intracellular pool of Aβ—*i*Aβ. Since the suppression of the production of Aβ in the AβPP proteolytic pathway (by verubecestat or other BACE1 inhibitors) had no effect on progression of the disease [49,50] either, it follows that in AD, Aβ is produced independently of AβPP and is retained intraneuronally. These two features, namely, the causative role of *i*Aβ in AD and its production and intracellular retention in the AβPP-independent pathway, are the major attributes of the ACH2.0 [1,2,3,4,5,6]. The conventional disease is triggered when the AβPP-derived *i*Aβ reaches ISR-activating levels, and it commences when the AβPP-independent *i*Aβ production pathway becomes operational [1,2,3,4,5,6]. The dynamics of accumulation of AβPP-derived *i*Aβ is, therefore, the deciding factor determining the occurrence of AD [4,6]. This is consistent with data showing that virtually all known FAD mutations accelerate the accumulation of AβPP-derived *i*Aβ and thus cause the early onset of the disease, and that the protective Icelandic mutation suppresses the accumulation of AβPP-derived *i*Aβ and thus delays or prevents the disease [4].

### 2.3. Origins of AβPP-Derived Intraneuronal Aβ: Two Physiologically Occurring Pathways

Conventionally, Aβ is derived from AβPP via two proteolytic cleavages. The first, by beta-secretase (beta-site AβPP-cleaving enzyme, BACE) occurs between residues 671 and 672 of AβPP and generates the C-terminal fragment (CTF) containing Aβ at its N-portion and consisting of 99 residues (designated C99). C99 is further cleaved, at variable positions, by gamma-secretase, thus generating Aβ with variable C-terminus and, accordingly, of variable length (typically 40 or 42 residues). Both cleavages occur on cellular membranes. The latter typically takes place on the plasma membrane and the resulting Aβ is secreted. Ostensibly, this scenario does not leave space for intraneuronal Aβ. The question is where it comes from. In fact, the intraneuronal Aβ is of two origins. First, the gamma cleavage of C99 does not occur exclusively on the plasma membrane. It also takes place within numerous cellular organelles. Those include lysosomes, endosomes, endoplastic reticulum, Golgi, trans-Golgi network, and mitochondria [74,75,76,77,78,79,80,81,82]. Importantly, Aβ resulting from C99 processing at these locations is not secreted outside the cell but is retained within it. The retention of Aβ produced on the intracellular membranes occurs physiologically and constitutes one origin of *i*Aβ. The second origin of intraneuronal Aβ is the importation—in fact, the repatriation—of secreted Aβ. The cellular uptake of extracellular Aβ also occurs physiologically [83,84,85,86,87,88], requires prior oligomerization of Aβ as a prerequisite [87,88], and is facilitated by a variety of cellular receptors [89,90,91,92,93,94,95,96,97].

### 2.4. Upon Reaching the Critical Threshold, iAβ Triggers the Activation of eIF2α Kinases, PKR and HRI, and the Elicitation of the ISR

When AβPP-derived *i*Aβ accumulates to a sufficient level (“the critical threshold”), it triggers the activation of two eIF2alpha kinases, namely, PKR and HRI. The link between Aβ and the PKR activity has been established in numerous studies [98,99,100] that showed not only the activated kinase but also the phosphorylated eIF2α in cells and model systems overexpressing Aβ. Importantly, the activated PKR was detected in neurons of Alzheimer’s patients [33,101]. It appears that *i*Aβ can activate PKR in two ways. One is via TNFα [26]. Another Aβ-mediated PKR activation pathway involves PKR ACTivator (PACT); its employment in AD is suggested by the observation of the co-localization of PACT and activated PKR in the neurons of AD patients [102].

The *i*Aβ-mediated activation of HRI in neuronal cells, on the other hand, is a consequence of the mitochondrial dysfunction. The connection between intracellular Aβ and mitochondrial dysfunction has been a subject of numerous investigation and is well established [103,104,105,106,107,108,109,110,111,112,113,114,115,116,117,118,119,120]. Mitochondrial distress has multiple ramifications for cellular physiology. One of the most important is the triggering of the integrated stress response. For the ISR to be elicited, a signal has to be conveyed from mitochondria to the cytosol. This involves two mitochondrial proteins. Fist, the mitochondrial distress activates the mitochondrial protease OMA1. OMA1, in turn, cleaves another mitochondrial protein, DELE1. One of the resulting fragments of DELE1 is exported to the cytosol. There, it binds to and activates the eIF2α kinase HRI [121,122], and the elicitation of the ISR follows. To summarize, the accumulation of AβPP-derived *i*Aβ to sufficient levels triggers, via two distinct pathways, the activation of two different eIF2α kinases, namely, PKR and HRI. As a result, eIF2α is phosphorylated at the serine residue 51 and the integrated stress response is elicited.

### 2.5. ISR-Reprogrammed Translation in Neuronal Cells Provides “Missing” Components of and Activates the AβPP-Independent iAβ Production Pathway: AD Commences

As discussed above, in conventional AD, the accumulation of *i*Aβ to the critical level triggers activation of the AβPP-independent *i*Aβ production pathway. The entire output of this pathway is retained within the cell; it drives the AD pathology, and the disease commences only when the pathway is activated [1,2,3,4,5,6]. What, apparently, leads to the activation of the AβPP-independent *i*Aβ generation pathway is the ISR. Under the ISR conditions, both transcription and translation are radically reprogrammed. The total protein production is severely suppressed but, concurrently, the translation of a small subset of cellular proteins is activated. Presumably [1,2,3,4,5,6], this subset includes the component(s), which are “missing” under regular conditions and are required for the activation and operation of the AβPP-independent *i*Aβ production pathway. When this/these component(s) become available, the pathway is activated and AD commences. It should be mentioned that whereas the end product of the AβPP-independent pathway is intraneuronally retained Aβ, *i*Aβ, its primary translation product is the C100 fragment of AβPP, i.e., the N-terminal methionine-containing C99; the etiology of C100 as well as its processing are described below.

### 2.6. Unconventional AD: The Disease Could Be Triggered via iAβ-Independent Elicitation of the ISR

In conventional AD, the elicitation of the ISR in neuronal cells and consequent activation of the AβPP-independent *i*Aβ production are mediated by AβPP-derived *i*Aβ. However, as long as the elicitation of the ISR is sufficient for the activation of the AβPP-independent *i*Aβ production pathway, this does not necessarily have to be the case. Potentially, the ISR can be elicited in neuronal cells by a variety of stressors capable of activating one or more of the four members of the family of eIF2α kinases. As soon as the ISR is elicited in neurons, the AβPP-independent *i*Aβ generation pathway would be activated and AD would commence. As described in the following subsection, the latter is, or eventually becomes, self-sustaining and its continuous operation is, therefore, independent from the initial ISR-eliciting stressor. AD driven by such a process would be unconventional. Both conventional and unconventional AD are identical in that they are driven by the same AβPP-independent *i*Aβ production pathway. They differ, however, in the cause that triggers the elicitation of the ISR in neuronal cells and consequently of the AβPP-independent *i*Aβ production pathway. In conventional disease, it is AβPP-derived *i*Aβ accumulated to sufficient levels; whereas in unconventional AD, it is any other stressor operating sufficiently long (or repeatedly) to allow *i*Aβ produced in the AβPP-independent pathway to accumulate over the critical threshold and for the pathway to become self-sustaining. Thus, unconventional AD may have multiple causes. These include, potentially, traumatic brain injury, chronic encephalopathy, chronic inflammation, and viral and bacterial infections.

### 2.7. iAβ Generated in the AβPP-Independent Pathway Drives the AD Pathology and Sustains and Perpetuates Its Own Production: The Engine That Drives AD

To recap the above discussion, the ACH2.0 envisions conventional AD as the process, which occurs in two stages. In the first stage, AβPP-derived *i*Aβ accumulates physiologically via two distinct mechanisms. One is the cellular uptake of secreted Aβ. Another is the intraneuronal retention of a fraction of Aβ produced by the processing of its immediate precursor, C99, on intracellular membranes within various cellular organelles. If and when the AβPP-derived *i*Aβ levels in neuronal cells reach and cross the critical threshold, the integrated stress response is elicited. The immediate cause of ISR elicitation is the phosphorylation of the eIF2α at the serine residue 51. This occurs, potentially, in two ways. One is the *i*Aβ-mediated activation of the PKR kinase via TNFα and/or the PKR activator PACT. Another way is through the *i*Aβ-triggered mitochondrial dysfunction and the associated activation of the mitochondrial protease OMA1. The activated OMA1 cleaves another mitochondrial protein, DELE1. One of the DELE1 fragments produced by the OMA1 cleavage is exported to the cytosol, where it binds to and activates the HRI kinase. When activated, either PKR or HRI, or both, phosphorylate eIF2α and the elicitation of the ISR ensues.

**Figure 1:**
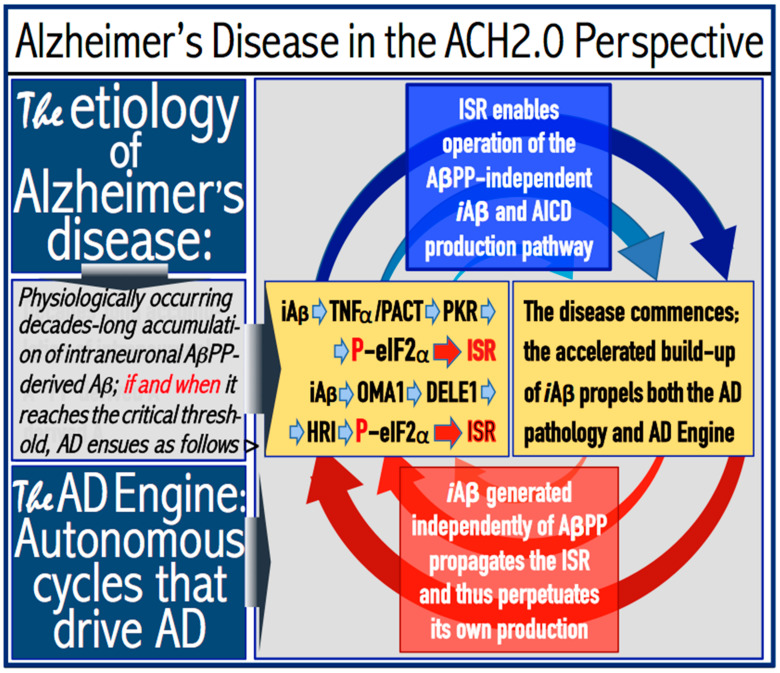
Conventional Alzheimer’s disease is caused by AβPP-derived *i*Aβ, accumulated physiologically over the ISR-triggering threshold, and is driven by *i*Aβ produced in the self-sustaining AβPP-independent pathway: the Engine that drives AD. *i*Aβ: intraneuronal Aβ. *eIF2α:* eukaryotic translation initiation factor 2alpha. *PKR*, *HRI:* kinases that phosphorylate eIF2α. *TNFα*: tumor necrosis factor/alpha (activates PKR); *PACT*: PKR activator. *OMA1:* mitochondrial protease activated by mitochondrial distress and cleaving DELE1; *DELE1:* mitochondrial protein mediating (after the cleavage by OMA1) the activation of HRI. *ISR:* integrated stress response, which is elicited by the phosphorylation of eIF2α and presumably activates the AβPP-independent *i*Aβ generation pathway. *AICD:* AβPP intracellular domain. *Gray box* (*left*): Physiologically occurring accumulation of *i*Aβ. If and when its levels reach the critical threshold, they trigger the activation of the PKR and/or HRI kinases. *Mustard-yellow box* (*left*)*:* Phosphorylation of eIF2α and the elicitation of the ISR; cellular protein synthesis is reprogrammed, resulting in the production of factor(s) required for the activation of the AβPP-independent *i*Aβ production pathway. *Blue box*: The AβPP-independent *i*Aβ generation pathway is initiated; its end products, *i*Aβ and AICD, are retained intraneuronally. *Mustard-yellow box* (*right*): Levels of *i*Aβ, driven by the AβPP-independent *i*Aβ production pathway, rapidly increase. *Red box:* High levels of *i*Aβ drive the AD pathology, sustain the activity of PKR and HRI, maintain the ISR conditions, and propagate the activity of the AβPP-independent pathway of its own production, thus perpetuating the operation of the AD Engine (arched blue and red arrows).

Under ISR conditions, cellular transcription and translation are reprogrammed and the global cellular protein synthesis is drastically suppressed. Concurrently, the translation of a small subset of cellular proteins, presumably in the cap-independent manner, is activated. Among those are the “missing” components required for the activity of the AβPP-independent *i*Aβ production pathway; when these components are made available, the pathway becomes operational. The entire *i*Aβ production output of the AβPP-independent pathway is retained within neuronal cells and its levels rapidly increase. This results in two major consequences. First, the elevated levels of *i*Aβ drive the AD pathology (AβPP-derived *i*Aβ cannot attain these levels), culminating in the formation of neurofibrillary tangles [123,124,125,126] and, ultimately, in the neuronal loss. Second, the resulting high levels of *i*Aβ sustain, via the continuous propagation of the ISR, its own production in the AβPP-independent pathway and consequently perpetuate its operation. Thus, in conventional AD, the ISR-eliciting stressor operating at the first stage of the disease (pre-ISR elicitation) is the same as the one operating at the second AD stage (post-ISR elicitation). In both cases, it is *i*Aβ, but of distinctly different origins. In the first AD stage, it is derived from AβPP via its proteolysis, whereas at the second stage it is produced independently of AβPP. When the AβPP-independent *i*Aβ production pathway is operational, it renders the influx of AβPP-derived *i*Aβ marginal and becomes fully independent from it. The repeated cycles of *i*Aβ-induced propagation of its own generation in the AβPP-independent pathway constitute the engine that powers AD—the AD Engine. The initiation and continuous operation of the AD Engine are depicted schematically in Figure 1.

## 3. Dynamics of *i*Aβ Accumulation and Its Role in AD in the ACH2.0 Perspective

### 3.1. iAβ Dynamics in Health and Disease

**Figure 2:**
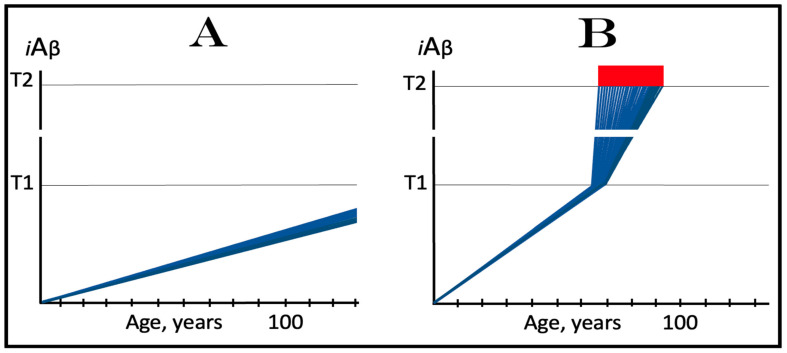
Dynamics of *i*Aβ accumulation in health and disease. *i*Aβ: intraneuronal Aβ. ***T1*** *threshold*: concentration of *i*Aβ that triggers activation of the eIF2α kinases, the elicitation of the ISR and the initiation of the AβPP-independent *i*Aβ generation pathway. ***T2*** *threshold*: concentration of *i*Aβ, generated mainly in the AβPP-independent pathway, which triggers neuronal apoptosis or necroptosis. *Blue lines*: levels of *i*Aβ in individual neuronal cells. *Red box*: apoptotic zone, the range of *i*Aβ levels that cause the commitment to cell death. (**A**) The levels of AβPP-derived *i*Aβ neither reach nor cross the T1 threshold within the lifetime of an individual; no disease occurs. (**B**) The levels of *i*Aβ cross the T1 threshold within a narrow temporal window; the ISR is elicited, and the AβPP-independent *i*Aβ generation pathway is initiated. AD commences only with the activation of the latter, and *i*Aβ produced independently of AβPP drives the AD pathology and simultaneously propels the operation of the AβPP-independent *i*Aβ production pathway.

In the ACH2.0, the dynamics of *i*Aβ accumulation in healthy individuals that do not develop AD in their lifetimes is single-phased. In these individuals, *i*Aβ is produced solely in the AβPP proteolytic pathway and accumulates, via its cellular uptake from the secreted extracellular pool and through the retention of a fraction generated by the processing of AβPP on the intracellular membranes, throughout the lifetime. As shown in panel A of Figure 2, its levels do not reach and never cross the threshold (T1 threshold), which triggers the activation of eIF2α kinases, the elicitation of the integrated stress response and the initiation of operation of the AβPP-independent *i*Aβ production pathway. Consequently, no AD occurs within the life span of an individual. It should be mentioned that if the T1 threshold is sufficiently high, the elevated, yet sub-T1, levels of *i*Aβ can cause aging-associated cognitive decline (AACD), a scenario that is outside the scope of the present study and that has been addressed elsewhere [4,6].

The dynamics of the *i*Aβ accumulation in AD-affected individuals, on the other hand, are two-phased. In the first phase, *i*Aβ is derived solely from AβPP. At this stage, the only difference from healthy individuals is that the rate of accumulation of AβPP-derived *i*Aβ is faster and/or the extent of the T1 threshold is lower, and it reaches and crosses the T1 threshold within the lifetime of an individual. In the second phase, the overwhelming bulk of *i*Aβ is generated independently of AβPP. Its levels rapidly increase, and when they reach and cross the T2 threshold, neurons commit apoptosis and/or necroptosis [127]. The two phases of the dynamics of *i*Aβ accumulation correspond to two stages of AD. The first AD stage is asymptomatic and culminates with the crossing of the T1 threshold [1,2,3,4,5,6]. The disease commences and its symptoms manifest at the second AD stage. At this stage, any interference with the production and/or accumulation of AβPP-derived *i*Aβ would have no effect whatsoever on the progression of AD because the disease is driven by *i*Aβ produced in the AβPP-independent pathway [1,2,3,4,5,6]. The dynamics of *i*Aβ accumulation in AD are presented diagrammatically in panel B of Figure 2.

### 3.2. Conditionality of the First AD Stage

Referring to the accumulation of AβPP-derived *i*Aβ prior to the crossing of the T1 threshold as “the first AD stage” creates a paradox. Indeed, there is obviously no AD at this stage. By definition, the disease commences and its symptoms manifest only with the activation of the AβPP-independent *i*Aβ production pathway at the second stage of AD. And if the T1 threshold were not crossed within the life span of an individual, a scenario currently predominating in the majority of general human population and illustrated in panel A of Figure 2, there would be no AD and, obviously, no first stage of it. Therefore, “the first stage of AD” is a conditional terminological construct. “The first AD stage” becomes such only post-factum, i.e., only if and when the T1 threshold is crossed, the AβPP-independent *i*Aβ production pathway activated, and the disease commences. Otherwise, the sub-T1 accumulation of AβPP-derived *i*Aβ is simply a normal physiological occurrence.

### 3.3. AD-Causing or -Preventing Mutations Act via the Augmentation or the Abatement of the Rate of Accumulation of AβPP-Derived iAβ

From the description of the dynamics of AβPP-derived *i*Aβ accumulation, it follows that it is the decisive factor, which determines the timing of the occurrence of the disease. The higher the rate of the AβPP-derived *i*Aβ accumulation is (and the lower the extent of the T1 threshold), the sooner the crossing of the T1 threshold occurs, AβPP-independent *i*Aβ production pathway become operational, and the disease commences. The slower the rate of AβPP-derived *i*Aβ accumulation (and the higher the extent of the T1 threshold), the later the T1 threshold is crossed and AD commences, and if the T1 is not reached within the life span of an individual, no AD occurs. These notions are supported by the observed effects of the mutations that either cause AD or protect from it. Not only the AD-causing mutations but also virtually all known factors that predispose to AD accelerate the kinetics of the AβPP-derived *i*Aβ accumulation. For instance, the internalization of extracellular Aβ requires ApoE. The latter can occur in several distinct isoforms. Of those, ApoE4 was shown to be more efficient in facilitating the cellular uptake of secreted Aβ than the rest of ApoE isoforms [66]. By increasing the influx of AβPP-derived *i*Aβ, it elevates the rate of its accumulation; it is also the major factor that predisposes its carriers to AD. In another example, certain PSEN mutations result in the shift of the gamma-cleavage to the position 42 of Aβ [85] and thus in the increased secretion of the Aβ42 isoform. Aβ42, in turn, is taken up by the cell twice as efficiently as the other Aβ isoforms [84]. Consequently, these mutations accelerate the rate of AβPP-derived *i*Aβ accumulation; they also cause the early onset of AD. Mutations resulting in the increased processing of AβPP on the intracellular membranes and, consequently, in the increased intraneuronal retention of AβPP-derived iAβ also increase the rate of its accumulation; they also cause the early onset of AD. This type of mutation is represented by the Swedish mutation [128] and some PSEN mutations [129]. The Flemish Aβ mutation lowers the efficiency of the physiologically occurring intra-*i*Aβ cleavages [130] and consequently increases the rate of accumulation of AβPP-derived *i*Aβ; it also causes the early onset of AD. The Icelandic Aβ mutation, on the other hand, elevates the efficiency of the physiologically occurring intra-*i*Aβ cleavage [59,60] and thus reduces the rate of the accumulation of AβPP-derived *i*Aβ; it also protects from AD (i.e., either delays or prevents it).

### 3.4. Potential Role of AICD in AD

The processing of C99 (or of C100, as described below), results in two products. Aβ is only one of those. Regardless of whether Aβ generated by the gamma-cleavage is secreted or retained within the neuron, the other product is always retained intraneuronally [131]. Therefore, it is designated the AβPP intracellular domain (AICD). In numerous studies, AICD was shown to be far from inert. Thus, it is known to interact with multiple cellular signaling pathways and to affect numerous regulatory proteins [132,133,134,135,136,137,138,139,140,141,142]. It was shown to participate in the regulation of gene expression and to affect both cytoskeletal dynamics and apoptosis [143,144]. It affects the *i*Aβ clearance by influencing the production of neprilysin [145], influences the phosphorylation of tau protein, and consequently the formation of NFTs [132,143]. It was also shown to impact the neuronal activity and oscillations in hippocampus, and to cause deterioration in spatial memory encoding [146]. AICD is generated both during the production of Aβ by AβPP proteolysis and during the operation of the AβPP-independent *i*Aβ production pathway. Therefore, there is substantially more AICD in AD patients than in healthy individuals. Due to its multiple activities, it is conceivable that it contributes significantly to AD pathology. The extent of its contribution, however, remains to be elucidated.

### 3.5. Inevitability of AD within Sufficiently Long Human Life Span

The majority of the general human population does not develop AD within their lifetime. What renders these individuals resistant to the disease is simply the slow kinetics of the accumulation of AβPP-derived *i*Aβ. No T1 threshold is crossed and consequently no AβPP-independent *i*Aβ production pathway is activated and no AD occurs within their life spans. The “life span” is the key term in the preceding statement; given the constant rate of accumulation of AβPP-derived *i*Aβ, it is the limited human life span that prevents the occurrence of AD. If, however, the life span is considered variable, with no limitation on its duration, the situation changes drastically: Everyone would eventually and inevitably develop AD provided her/his life span is sufficiently long. Indeed, since the accumulation of AβPP-derived *i*Aβ is, apparently, a lifelong process, it is only a question of time when its levels would cross the T1 threshold, but the crossing would certainly occur and AD develop. As the average life span steadily increases, so does the fraction of the general population that develops AD, and one can safely predict that this trend would continue, with the affected fraction nearing 100% unless a preventive treatment is developed and is implemented routinely [1,2,3,4,5,6].

## 4. AβPP-Independent Production of Intraneuronally Retained Aβ Is Inoperative in the Current Transgenic Animal Models of AD: *i*Aβ Dynamics in the ACH2.0 Perspective

### 4.1. In the Current Transgenic AD Models, Levels of AβPP-Derived iAβ Cross the T1 Threshold and Elicit the ISR

In the ACH2.0 perspective, the observed limited symptoms of AD in the current transgenic models of the disease are caused mainly by the ISR triggered by intraneuronal AβPP-derived *i*Aβ accumulated over the critical threshold. As in humans, the latter has two origins. One is the cellular uptake of secreted Aβ, and another the intraneuronal retention of a fraction of AβPP-derived Aβ following the gamma-cleavage on the intracellular membranes. Both processes occur physiologically and both are enhanced by the acute overproduction of AβPP from multiple transgenes. As discussed below, at sufficient cellular concentrations, *i*Aβ is a stressor capable of eliciting the ISR. If and when the levels of AβPP-derived *i*Aβ cross the T1 threshold, they would trigger the activation of the PKR and/or HRI kinases, phosphorylation of eIF2α, and elicitation of the integrated stress response. There are strong indications that the above sequence of events indeed takes place and that the ISR is elicited in transgenic AD models. As described in the preceding sections, the ISR was shown to cause cognitive impairments such as defects in the neuronal plasticity, long-term memory formation, and learning, processes that require de novo protein synthesis, which is suppressed under ISR conditions. That the observed cognitive deficits in transgenic AD models are caused by the ISR was convincingly demonstrated in studies utilizing the small-molecule ISR inhibitor ISRIB as well as genetic prevention and pharmacological suppression of the ISR. Indeed, in these studies, inhibition of the ISR or the prevention of its elicitation resulted in the marked alleviation or in the preclusion of cognitive impairments in the current transgenic AD models [37,38,39,40,41,42,43,44,45].

### 4.2. Inactivity of the AβPP-Independent iAβ Production Pathway in Transgenic AD Models Defines the Single-Phased Dynamics of Its Accumulation

**Figure 3:**
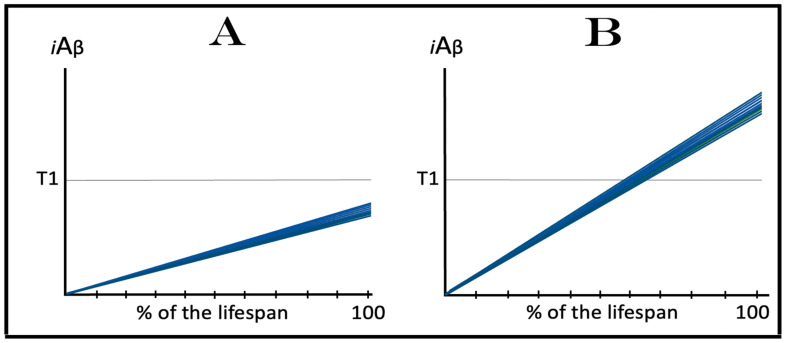
*i*Aβ dynamics in normal mice and in the current transgenic models of AD: the ACH2.0 perspective. *i*Aβ: intraneuronal Aβ. ***T1*** *threshold*: concentration of *i*Aβ that triggers activation of the eIF2α kinases, phosphorylation of eIF2α, and the elicitation of the ISR. *Blue lines*: levels of *i*Aβ in individual neuronal cells. (**A**) In normal mice, the levels of AβPP-derived *i*Aβ neither reach nor cross the T1 threshold within the lifetime of an animal. (**B**) Aβ is produced from multiple transgenes, and the rate of the accumulation of AβPP-derived *i*Aβ markedly increases. The levels of AβPP-derived *i*Aβ cross the T1 threshold, and the ISR is elicited. The resulting transcriptional and translational reprogramming and severe suppression of the total protein production cause impairment in cognitive functions requiring de novo protein synthesis. The AβPP-independent *i*Aβ generation pathway is not activated; no AD occurs. *i*Aβ is produced throughout the lifetime solely in the AβPP proteolytic pathway, and its rate of accumulation does not change.

In conventional AD, the AβPP-derived *i*Aβ-mediated elicitation of the integrated stress response leads to the activation of the AβPP-independent *i*Aβ production pathway. Since the entire *i*Aβ output of this pathway is retained within neuronal cells, its levels rapidly increase. High levels of *i*Aβ, produced overwhelmingly in the AβPP-independent pathway, appear to be essential for both the commencement of the disease and the progression of the AD pathology [1,2,3,4,5,6]. In the current transgenic animal models of AD, as reasoned above, AβPP-derived *i*Aβ accumulates over the T1 threshold and the integrated stress response is elicited, but the AD pathology does not progress, judging by its major hallmark, the formation of neurofibrillary tangles, or rather by the lack thereof; indeed, there are no indications that the disease commences in the first place. It follows that in the current transgenic AD models, the elicitation of the ISR is not accompanied by the enhanced production and accumulation of *i*Aβ, i.e., that in transgenic AD models the elicitation of the ISR is not followed by the activation of the AβPP-independent *i*Aβ production, which remains inoperative. Under these circumstances, the AβPP proteolytic pathway would remain the only source of *i*Aβ and its accumulation would continue at the same rate as prior to the crossing the T1 threshold. Such dynamics of the *i*Aβ accumulation in the current transgenic AD models are illustrated diagrammatically in Figure 3. The figure shows two conditions. In normal (non-transgenic) mice, AβPP-derived *i*Aβ accumulates slowly, and neither crosses the T1 threshold nor causes the elicitation of the ISR and accompanying cognitive impairment within the lifetime of an animal. In contrast, in transgenic animals, the rate of the accumulation of AβPP-derived *i*Aβ is increased due to its massive overproduction from multiple transgenes, and, consequently, the T1 threshold is crossed; the ISR is elicited, and cognitive impairment manifests. However, with the AβPP-independent *i*Aβ production pathway inoperative, *i*Aβ does not reach, within the limits of the life span of an animal, levels essential to support the progression (and, apparently, even the commencement) of AD pathology and formation of NFTs. Since we define AD as a disease that initiates with the activation of the AβPP-independent *i*Aβ production pathway (see above), it neither commences nor occurs in the current transgenic animal AD models. These models are useful in many respects; they, however, are not AD models.

## 5. Why ACH-Based AD Drugs Are Effective in Current Transgenic AD Models, but Not in Symptomatic AD Patients

### 5.1. Effect of ACH-Based Drugs in Current Transgenic AD Models

**Figure 4:**
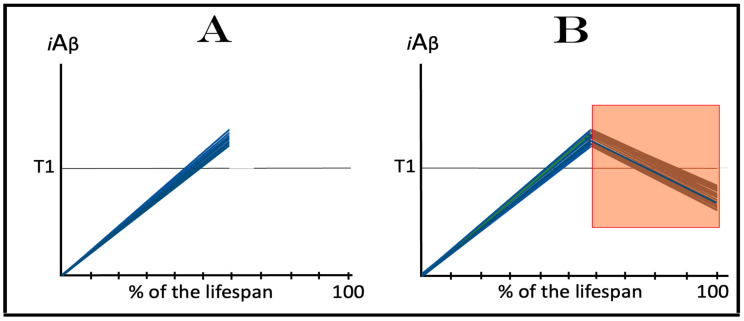
Effect of ACH-based drugs in the current transgenic animal AD models. *i*Aβ: intraneuronal Aβ. ***T1*** *threshold*: concentration of *i*Aβ that triggers activation of the eIF2α kinases, phosphorylation of eIF2α, and the elicitation of the ISR. *Blue lines*: levels of *i*Aβ in individual neuronal cells. *Orange Box:* duration of the administration of the ACH-based drug. (**A**) The initial state of the levels of *i*Aβ in the individual neurons at the commencement of drug’s administration. The levels of AβPP-derived *i*Aβ have crossed the T1 threshold. The ISR has been elicited, and the ISR-caused cognitive impairments (due to the suppression of cellular protein synthesis) have manifested. (**B**) Evolution of the initial state in the presence of the ACH-based drug (for the definition of ACH-based drugs and their mechanism of action, see the main text). The drug reduces the influx of *i*Aβ and consequently lowers its rate of accumulation. In the best-case scenario (shown in the Figure), due to the physiologically ongoing *i*Aβ clearance, its rate of accumulation is reversed. When the levels of *i*Aβ are reduced below the T1 threshold, the ISR is no longer in effect; the normal protein synthesis is restored and the cognitive impairment is relieved for the duration of the drug’s administration.

The dynamics of the accumulation of AβPP-derived *i*Aβ in the current transgenic animal models of AD, described in the preceding section, explain why ACH-based AD drugs are so effective in these models. ACH-based drugs were designed to reduce levels of extracellular Aβ. They can be divided into two categories. One consists of drugs that either degrade extracellular Aβ or sequester it, as in the cases of numerous monoclonal antibodies. Another category includes drugs that suppress the production and consequently secretion of Aβ in the AβPP proteolytic pathway. These drugs are exemplified by various BACE1 inhibitors, such as verubecestat. In the ACH2.0 perspective, these drugs should also be effective (see limitations below), because by reducing the pool of extracellular Aβ, they also reduce the rate of its uptake into the cell. Moreover, the second category of ACH-based drugs reduce not only its importation but also its intraneuronal retention (less produced, less retained). When implemented, such drugs would reduce the rate of AβPP-derived *i*Aβ accumulation and could even reverse it due to the physiologically ongoing *i*Aβ clearance; this would obviously be therapeutically beneficent. The expected, and apparently observed, effect of ACH-based drugs in current transgenic animal AD models is illustrated in Figure 4. The drug is administered when the T1 threshold has already been crossed, the ISR elicited, and cognitive impairment manifested. For the duration of drug administration, the rate of *i*Aβ accumulation is reversed. When its levels are reduced below the T1 threshold, the ISR is no longer in effect, the normal protein synthesis is restored and the cognitive impairment is relieved. The key to the drugs’ efficiency in transgenic animal models is the inactivity of the AβPP-independent *i*Aβ production pathway, which is insensitive to these drugs.

### 5.2. Effect of ACH-Based Drugs in Symptomatic AD

**Figure 5:**
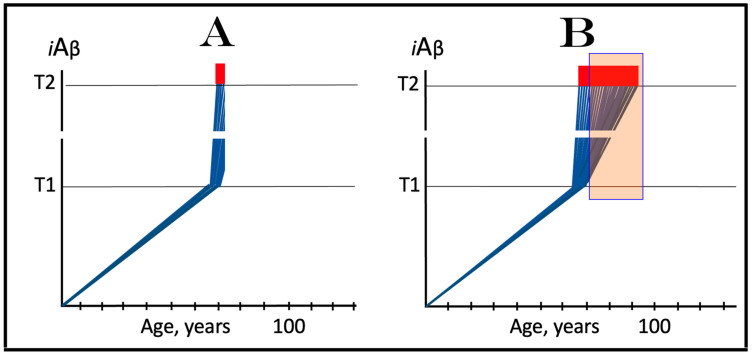
Effect of ACH-based drugs in symptomatic Alzheimer’s disease. *i*Aβ: intraneuronal Aβ. ***T1*** *threshold*: concentration of *i*Aβ that triggers activation of the eIF2α kinases, the elicitation of the ISR and the initiation of the AβPP-independent *i*Aβ generation pathway. ***T2*** *threshold*: concentration of *i*Aβ, generated mainly in the AβPP-independent pathway, which triggers neuronal apoptosis or necroptosis. *Blue lines*: levels of *i*Aβ in individual neuronal cells. *Red Box*: apoptotic zone, the range of *i*Aβ levels that cause the commitment to cell death. *Orange Box:* duration of the administration of the ACH-based drug. (**A**) The initial state of the levels of *i*Aβ in the individual neurons at the commencement of drug administration. The levels of AβPP-derived *i*Aβ have crossed the T1 threshold. The ISR has been elicited, and the AβPP-independent *i*Aβ production pathway was activated in all affected neurons. A fraction of the affected neurons have crossed the T2 threshold, committed apoptosis, and AD symptoms have manifested. (**B**) Evolution of the initial state in the presence of the ACH-based drug. The drug reduces the influx and lowers the rate of accumulation of AβPP-derived *i*Aβ. At this stage, however, *i*Aβ is overwhelmingly produced in the AβPP-independent pathway. The contribution of the AβPP-derived *i*Aβ into the cellular *i*Aβ pool is marginal, and its suppression is inconsequential and has no impact whatsoever on the progression of AD. *i*Aβ continues to accumulate, its levels cross the T2 threshold, and when a sufficient fraction of the neurons commit apoptosis, the disease enters the end stage.

In the framework of the ACH2.0, the outcome of the implementation of ACH-based drugs in symptomatic AD patients is expected to be, and evidently was, drastically different. The key to this difference is the operational AβPP-independent *i*Aβ production pathway. As shown in Figure 5, at the time of drug administration, levels of AβPP-derived *i*Aβ have crossed the T1 threshold and the AβPP-independent *i*Aβ production pathway has been activated in all affected neurons [1,2,3,4,5,6]. At this stage, *i*Aβ is overwhelmingly produced in the AβPP-independent pathway. Whereas the activation of this pathway is triggered by AβPP-derived *i*Aβ, its operation is completely independent of the latter. Indeed, the AβPP-independent *i*Aβ production pathway is self-sustaining because its *i*Aβ product propagates its own generation (this is the reason why it is referred to as the AD Engine) and also drives the AD pathology. On the other hand, with the AβPP-independent *i*Aβ production pathway operative, the contribution of the AβPP proteolytic pathway to the cellular *i*Aβ pool becomes marginal and inconsequential for the progression of the disease. ACH-based drugs can, and apparently do, interfere with the accumulation of AβPP-derived *i*Aβ, but they cannot affect the production or accumulation of *i*Aβ in the AβPP-independent pathway. Therefore, the implementation of ACH-based drugs would be futile in symptomatic AD, as was indeed observed in clinical trials.

### 5.3. ACH-Based Drugs Would Be Effective in Prevention of AD for the Same Reason They Are Effective in Transgenic AD Models

**Figure 6:**
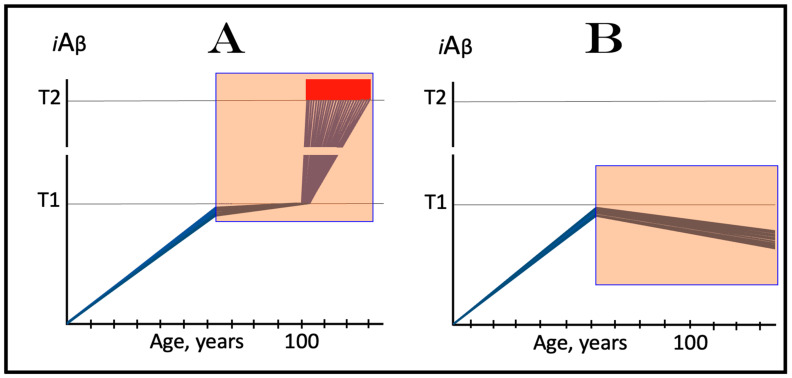
Effect of ACH-based drugs in prevention of AD. *i*Aβ: intraneuronal Aβ. ***T1*** *threshold*: concentration of *i*Aβ that triggers activation of the eIF2α kinases, the elicitation of the ISR and the initiation of the AβPP-independent *i*Aβ generation pathway. ***T2*** *threshold*: concentration of *i*Aβ, generated mainly in the AβPP-independent pathway, which triggers neuronal apoptosis or necroptosis. *Blue lines*: levels of *i*Aβ in individual neuronal cells. *Red box*: apoptotic zone, the range of *i*Aβ levels that cause the commitment to cell death. *Orange boxes:* duration of the administration of the ACH-based drug. The drug is administered prior to the crossing of the T1 threshold. At this stage, the AβPP-independent *i*Aβ production pathway is inoperative and *i*Aβ is derived solely from AβPP via its proteolysis. Therefore, the interference with its accumulation would delay or prevent the crossing of the T1 threshold and the commencement and indeed the occurrence of AD. (**A**) The rate of the accumulation of AβPP-derived *i*Aβ is reduced, but its levels continue to increase. Eventually, they would reach and cross the T1 threshold. The ISR would be elicited, the AβPP-independent *i*Aβ production pathway would be activated, and AD would commence, but all this would occur with a considerable delay in comparison to an untreated individual. (**B**) The influx of AβPP-derived *i*Aβ is reduced sufficiently to reverse the rate of its accumulation. Consequently, no T1 would be crossed, no AβPP-independent *i*Aβ production pathway would be activated, and no AD would occur for the duration of the treatment.

On the other hand, in the ACH2.0 perspective, ACH-based drugs could potentially be effective in prevention of AD, if administered prior to the commencement of the disease, for exactly the same reason they are effective in transgenic AD models: the inactivity of the AβPP-independent *i*Aβ production pathway at this stage. Preventive implementation of ACH-based drugs infers that they are administered prior to the crossing of the T1 threshold. At this stage, *i*Aβ is derived solely from AβPP via its proteolysis. Therefore, interference with its accumulation either through the reduction of the rate of its importation from the extracellular pool or via the suppression of its intraneuronal retention by the inhibition of its production would delay or prevent the crossing of the T1 threshold and the commencement and indeed the occurrence of AD. This expected preventive effect of ACH-based drugs is shown in Figure 6. In panel A, the rate of the accumulation of AβPP-derived *i*Aβ is reduced but its levels continue to increase, albeit more slowly. Eventually, they would reach and cross the T1 threshold. The ISR would be elicited, the AβPP-independent *i*Aβ production pathway would be activated, and AD would commence, but all this would occur with a considerable delay in comparison to an untreated individual. In panel B, the influx of AβPP-derived *i*Aβ is reduced sufficiently to reverse the rate of its accumulation. Consequently, no T1 would be crossed, no AβPP-independent *i*Aβ production pathway would be activated, and no AD would occur for the duration of the treatment.

### 5.4. ACH-Based Drugs Can Be Only Marginally Effective in Early Symptomatic AD: Effects of Lecanemab and Donanemab, the Proverbial Exceptions That Prove the Rule

**Figure 7:**
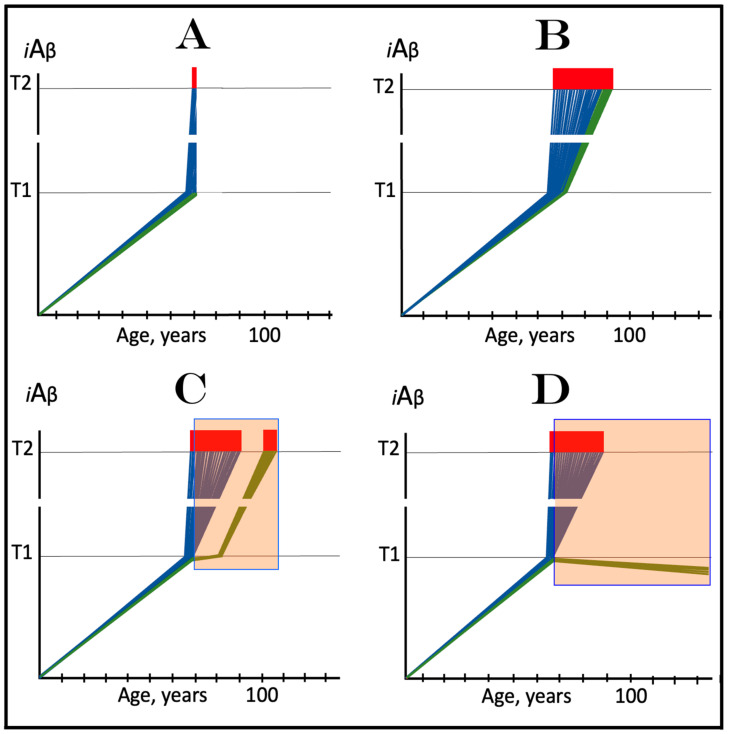
Effect of ACH-based drugs in early symptomatic AD: lecanemab and donanemab. *i*Aβ: intraneuronal Aβ. ***T1*** *threshold*: concentration of *i*Aβ that triggers activation of the eIF2α kinases, the elicitation of the ISR and the initiation of the AβPP-independent *i*Aβ generation pathway. ***T2*** *threshold*: concentration of *i*Aβ, generated mainly in the AβPP-independent pathway, which triggers neuronal apoptosis or necroptosis. *Blue lines*: levels of *i*Aβ in individual neuronal cells. Green lines: neurons with levels of AβPP-derived *i*Aβ below the T1 threshold at the commencement of the treatment. *Red box*: apoptotic zone, the range of *i*Aβ levels that cause the commitment to cell death. *Orange boxes:* duration of the administration of the ACH-based drug. (**A**) The initial state of *i*Aβ levels in individual neurons at the commencement of the drug’s administration. The levels of AβPP-derived *i*Aβ have crossed the T1 threshold and the AβPP-independent *i*Aβ production pathway has been activated in the bulk of the neurons, but a small neuronal fraction remains sub-T1. (**B**) Evolution of the initial state in the absence of a treatment. The initial sub-T1 neuronal fraction crosses the T1 threshold and activates the AβPP-independent *i*Aβ production pathway. The disease progresses until it reaches the end stage. (**C**,**D**) Evolution of the initial state in the presence of the drug. In both panels, the drug affects only the sub-T1 neuronal fraction and has no effect in neurons with the operational AβPP-independent *i*Aβ generation pathway. (**C**) In the initial sub-T1 neuronal fraction, the drug reduces the influx of AβPP-derived *i*Aβ and lowers the rate of its accumulation, but its levels are increasing and eventually reach the T1 threshold. The AβPP-independent *i*Aβ production pathway would be activated, the cells would become unresponsive to the drug, and their fate would be the same as of the rest of the neuronal population, albeit with a certain delay. (**D**) The influx of AβPP-derived *i*Aβ is reduced sufficiently to reverse its accumulation, and its levels are decreasing. In this neuronal fraction, the T1 threshold would not be reached for the duration of the drug’s administration. In either case, the overall effect would be only marginal because the drug would affect only a marginal neuronal sub-population.

The preventive potential of ACH-based drugs in AD explains both the nature of their observed effect at the very early stages of the disease, as was seen in the recent clinical trials of lecanemab and donanemab, and why this effect was only marginal. In contrast to the preceding clinical trials of potential AD drugs, which utilized participants at relatively advanced stages of AD, in the clinical trials in question [147,148,149,150,151], only subjects at the very early stages of the disease were employed. As described above and elsewhere [1,2,3,4,5,6], in AD patients, the levels of AβPP-derived *i*Aβ in individual affected neurons cross the T1 threshold, and thus initiate the disease, within a narrow temporal window. Consequently, when AD symptoms manifest, the bulk if not the entire population of the affected neurons have crossed the T1 threshold and activated the AβPP-independent *i*Aβ production pathway. As reasoned above, the implementation of ACH-based drugs at this point would be futile. As shown in Figure 7, in the clinical trials of lecanemab and donanemab, however, at the time of the drug administration, due to the early stages of the disease, a fraction of the affected neurons in individual subjects have not yet crossed the T1 threshold and therefore were responsive to the drug. The beneficial effect of the drags in these clinical trials was thus preventive, not curative. It was marginal because the fraction of sub-T1 neurons was marginal. It should be emphasized that there is nothing special about lecanemab and donanemab. What made the difference (in comparison with the preceding trials) was the early timing of their administration. Any typical ACH-based drug, administered at the same early stage of AD, would have similar effect. Thus, whereas the preventive implementation of ACH-based AD drugs could be feasible, this is, apparently, not the case in symptomatic AD patients.

## 6. AβPP-Independent Production of *i*Aβ Is the Cornerstone of Any Adequate Model of AD

The AβPP-independent *i*Aβ production pathway appears to constitute the essence, the active core of AD. Indeed, the accumulation of *i*Aβ produced in the AβPP proteolytic pathway alone appears insufficient to reach the levels required to initiate and drive the disease. The efficiency of the AβPP-independent *i*Aβ generation pathway greatly exceeds that of its production in the AβPP proteolytic pathway. The reasons for this are multiple. Whereas only a minute fraction of Aβ produced by AβPP proteolysis ends up as *i*Aβ, the entire output the AβPP-independent *i*Aβ production pathway is, presumably, retained intraneuronally. The primary translation product of the AβPP-independent pathway of *i*Aβ generation (100 amino acid residues long) constitutes only 13% of the full-size AβPP (771 residues long) and requires only one, rather than two, proteolytic cleavage; accordingly, its production is an order of magnitude more efficient. Moreover, as discussed in detail below, it appears plausible that the AβPP-independent *i*Aβ generation pathway is powered by the asymmetric amplification of AβPP mRNA. In this scenario, every conventionally transcribed AβPP mRNA serves repeatedly as a template for transcription of multiple mRNAs encoding C100 [4], and the rate of its production in such a case would be orders of magnitude greater than that of C99 production by AβPP proteolysis. In relation to the AβPP-independent *i*Aβ production pathway, AβPP-derived *i*Aβ plays an auxiliary role: just as the starter motor ignites the car engine (and remains redundant for the duration of the engine’s operation), so too does AβPP-derived *i*Aβ, when accumulated over the T1 threshold, ignite the autonomous, self-sustaining AD Engine and is rendered marginal, if not redundant, afterwards. The bottom line of this reasoning is that the operation of the AβPP-independent *i*Aβ production pathway is necessary and probably sufficient for AD; the disease cannot occur without it. This pathway, therefore, is the cornerstone of and has to be incorporated in any adequate model of the disease.

## 7. Human Neuronal Cell-Based Models of AD

The present section describes the design, construction, and utilization of the human neuronal cell-based AD models capable of displaying the full spectrum of cellular AD pathology. These models are sufficient to address numerous aspects of the disease and to support the development and testing of novel AD drugs. Importantly, they also constitute the essential intermediate step in the development of adequate transgenic animal models of AD.

### 7.1. Rationale

Conceivably, for more than one reason, the best and apparently the only currently available adequate model of AD is one based on human neuronal cells. AD appears to be a human-specific condition. It is possible that it occurs in other species, but so far it has been observed exclusively in humans (all claims to the contrary have been made on the basis of the appearance of Aβ plaques, a criterion that has little relevance to AD in the ACH2.0 perspective [1,2,3,4,5,6]). Closely related primate species possibly do not live long enough to develop the disease, but even in long-lived mammals such as elephants, no AD has been detected. Since the ACH2.0 defines AD as a disease driven by the production of *i*Aβ in the AβPP-independent pathway, it can be assumed that this attribute is possibly unique (or at least relatively unique) to humans and is inoperative (or operative rarely) in non-human mammalian species. Thus, choosing human neuronal cells as the basis for the development of AD model confers two advantages. One, these cells originate from the species known to be affected by the disease. Another, related, advantage is that it can be presumed that they are capable of operating molecular pathways underlying the disease; more specifically that they are capable, when properly induced, to generate *i*Aβ in the AβPP-independent pathway. Given this capability, the design of the human neuronal cell-based model of AD is obvious: activate the AβPP-independent *i*Aβ production pathway and ascertain that it is self-sustaining, i.e., that the AD Engine is operative, and the cellular AD pathology would follow; the activity of the AβPP-independent *i*Aβ generation pathway can be assessed as described in Section 12 below. At this point, the cellular AD pathology would become, short of therapeutic intervention, irreversible and the appearance of neurofibrillary tau tangles could serve as the benchmark for the occurrence and the progression of the disease at the cellular level.

### 7.2. Exogenous iAβ-Mediated Elicitation of the ISR

According to the ACH2.0, the activation of the AβPP-independent *i*Aβ production pathway is preceded by the elicitation of the ISR: the latter causes the former. One way to elicit the ISR is via the accumulation of exogenous *i*Aβ. Such an approach would emulate the physiological development of conventional AD. Presumably, once the levels of *i*Aβ reach the T1 threshold, the PKR and/or HRI kinases would be activated, eIF2α would be phosphorylated, the ISR would be elicited and the operation of the AβPP-independent pathway of production of endogenous *i*Aβ would be initiated. Exogenous AβPP can be produced transiently or stably, from multiple AβPP-encoding transgenes. To prevent the diffusion of secreted Aβ (and consequent reduction of its importation), cells can be maintained in a semi-solid medium such as Matrigel. Utilization of the proper AβPP mutants would accelerate the accumulation of AβPP-derived *i*Aβ. Thus, mutants producing predominantly Aβ42 would elevate the rate of its cellular uptake and also lower the extent of the T1 threshold [4]. Utilization of the Swedish AβPP mutant, in another example, would result, as discussed above, in the increased rate of intraneuronal retention of Aβ produced on the intracellular membranes.

In another approach, *i*Aβ can be produced exogenously from vectors or from transgenes expressing only Aβ42. In this approach, its entire output would remain within the cell (it lacks the transmembrane domain, which is present within C99 at the junction of its Aβ and AICD segments and is requisite for secretion) and would rapidly accumulate. When its levels cross the T1 threshold, the ISR would be elicited and the AβPP-independent endogenous production of *i*Aβ would be activated. Since at the time of the activation of the AβPP-independent *i*Aβ production pathway, the basal *i*Aβ level would be above the T1 threshold, operation of the pathway would be self-sustainable as soon as it is active.

### 7.3. iAβ-Independent Elicitation of the ISR

In the framework of the ACH2.0, elicitation of the ISR in human neuronal cells by means other than AβPP-derived *i*Aβ would be sufficient to activate the endogenous AβPP-independent *i*Aβ production pathway and to trigger the progression of the AD pathology. This is, in all likelihood, the way in which traumatic brain injury, chronic encephalopathy, chronic inflammation, and viral and bacterial infections contribute to the development of AD. To trigger the elicitation of the ISR in human neuronal cells, it is sufficient to activate any of the four eIF2α kinases: PKR, PERK, GCN2, and HRI. There are numerous stressors capable of activating these kinases. For example, HRI could be conveniently activated via mitochondrial disorder as was described for various cell types, including neuronal cells [121,122]. In this approach, however, when the ISR is elicited and the endogenous AβPP-independent *i*Aβ production pathway is activated, the basal level of *i*Aβ would be below the T1 threshold. Consequently, at the time of its activation, the AβPP-independent *i*Aβ production pathway wouldn’t be self-sustainable, and if the initial ISR-eliciting stressor is removed and the ISR is not in effect anymore, operation of the pathway would cease. It follows that in this approach the initial stressor should be present, to maintain the ISR, long enough to allow *i*Aβ produced in the AβPP-independent pathway to accumulate over the T1 threshold. At this level, *i*Aβ becomes the stressor, which maintains the ISR (via activation of the PKR and/or HRI kinases) and perpetuates the operation of the AβPP-independent pathway of its own production; the continuous presence or the removal of the initial ISR-eliciting stressor would be, at this point, irrelevant and inconsequential for operation of the pathway.

### 7.4. Proof of Concept: “Alzheimer’s in the Dish”

As was discussed above, the appearance of neurofibrillary tau tangles can serve as the benchmark for the progression of the cellular AD pathology in human neuronal cell-based AD models. But are cultured human neuronal cells capable of displaying the full spectrum of cellular AD pathology, including the NFTs? The answer to this question is affirmative. The formation of NFTs was indeed observed in human neuronal cells overexpressing exogenous Aβ and cultured in Matrigel [152] (a model popularly known as “Alzheimer’s in the dish”). The authors of that study interpreted (in the ACH terms) the results as the affirmation of the AD-causing effect of extracellular Aβ. The interpretation of these results in the ACH2.0 framework, however, indicated that in this study, the endogenous self-sustaining AβPP-independent *i*Aβ production pathway was activated and that this pathway propelled the cellular AD pathology, including the formation of the NFTs. In the study in question, a polycistronic lentiviral construct was employed to overexpress human AβPP carrying two FAD mutations, namely, London (V717I) and Swedish (K670N/M671L), as well as PSEN1 with the E9 FAD mutation. The construct was transfected into human neural progenitor cells, which were cultured and differentiated in Matrigel. In the resulting neuronal cells, the Swedish mutation promoted, as discussed above, the processing of C99 on intracellular membranes and the retention of *i*Aβ. The London mutation shifted the AβPP processing toward production of the Aβ42 isoform, as did the PSEN1 E9 mutation. Since, as discussed above, the rate of importation of extracellular Aβ42 (which did not diffuse due to the cultivation of cells in Matrigel) is twice that of other Aβ isoforms, and because of the increased retention of AβPP-derived *i*Aβ produced on intracellular membranes, the rate of accumulation of exogenous *i*Aβ significantly accelerated; eventually it crossed the T1 threshold and triggered the activation of the endogenous AβPP-independent *i*Aβ production pathway. With this pathway operative, the cellular AD pathology progressed and reached the benchmark of the NFTs formation.

The study under discussion [152] serves as proof of concept for the suitability of human neuronal cells as the basis for the models of AD. As described above and elsewhere [1,4], the design of human neuronal cell-based models of AD can be significantly streamlined, but the model utilized in [152] can also be legitimately employed in further studies.

### 7.5. Human Neuronal Cell-Based AD Model as a Tool for Validation of the Occurrence of AβPP-Independent iAβ Production and for Elucidation of the Molecular Nature of the Underlying Mechanism

Above, we reasoned that to generate the adequate transgenic animal model of AD, the operative inducible AβPP-independent *i*Aβ production pathway has to be introduced. The problem is that we do not know the identity of this pathway and need an adequate AD model in order to test for it. Human neuronal cell-based AD models solve this problem. With such a model, we do not have to know the nature of the mechanism, which produces *i*Aβ independently of AβPP, in order to employ the model. It is sufficient to know that it is incorporated into the model. And because the model is based on human neuronal cells, we are certain that it is, intrinsically. The availability of such an AD model provides a tool for validation of operation of the AβPP-independent *i*Aβ generation pathway and for elucidation of its molecular nature. In Section 12 below, we describe how the operation of this pathway can be verified. We also define four distinct mechanisms capable of generating *i*Aβ independently of AβPP and describe how they can be tested for and identified with the help of human neuronal cell-based AD models (Section 13 below).

### 7.6. Human Neuronal Cell-Based AD Model as a Tool for Testing Novel AD Drugs

Human neuronal cell-based models of AD can also be employed to evaluate potential therapeutic effects of the novel AD strategies, such as, for example, the depletion of *i*Aβ by its targeted degradation via the activation of BACE1 and/or BACE2. The rationale for this strategy, described in detail in [4,6], is, briefly, the following. The AβPP-derived *i*Aβ production pathway, which drives the AD pathology, is self-sustainable. It is propagated by *i*Aβ at the levels above the T1 threshold. If *i*Aβ were depleted to the levels below the T1 threshold, operation of the pathway would cease and the progression of the AD pathology would be arrested. This can be achieved by the transient activation of BACE1 and/or BACE2. Both possess intra-*i*Aβ cleaving activities (distinctly different; secondary in BACE1 and primary in BACE2) that are capable of depleting *i*Aβ if sufficiently enhanced (reviewer in [4,6]; in fact, this is what takes place in carriers of the protective Icelandic Aβ mutation). The disease would not recur until the levels of *i*Aβ (now produced solely in the AβPP proteolytic pathway) would be restored to the T1 (i.e., the ISR-activating) threshold, possibly a decades-long process. To evaluate their therapeutic potential, BACE1 and/or BACE2 can be exogenously overexpressed in human neuronal cell-based AD model. Assaying for the effects of BACE1/2 overexpression would include monitoring *i*Aβ levels, expected to be reduced, measuring the activity of the AβPP-independent *i*Aβ generation pathway (expected to cease if the strategy is successful), as described in Section 12 below, and testing for the occurrence of NFTs.

Human neuronal cell-based AD models can also be employed to evaluate the feasibility of the ISR inhibitors as potential AD drugs. Indeed, in the ACH2.0 paradigm, if the ISR is prevented or suppressed, the AβPP-independent *i*Aβ production pathway cannot operate, and consequently AD cannot occur (or progress). The means to inhibit the integrated stress response are currently available: the small-molecule ISR inhibitor ISRIB. Depending on the timing of its administration and on the duration of treatment, it could be anticipated that the implementation of ISRIB would either prevent both the activation of the AβPP-independent *i*Aβ production pathway and the formation of neurofibrillary tangles (if dispensed prior to the elicitation of the ISR) or stop operation of the former (if applied following the elicitation of the ISR; approaches to assess the activity of this pathway are discussed below). If successful, the results of such assessment would establish the ISR inhibitors as potential AD drugs. It should be mentioned that the utilization of the ISR inhibitors as AD drugs would require the long-term duration of treatment, which could be problematic in view of the pivotal physiological role of the ISR.

## 8. Potential Mechanisms Enacting AβPP-Independent *i*Aβ Production in AD: The Singularity of the AUG Encoding Met671 of Human AβPP

### 8.1. Pivotal Role of the AUG Encoding Met671 of AβPP in the AβPP-Independent Generation of iAβ

All conceivable mechanisms potentially underlying operation of the AβPP-independent *i*Aβ production pathway have one common feature: in all, translation initiates from the AUG conventionally encoding methionine 671 of human AβPP [4]. This pivotal role of the AUG codon in question stems from its singular position within the human AβPP gene, and, consequently, within AβPP mRNA. In 1987, three research groups cloned and sequenced human AβPP cDNA [153,154,155]. Shortly afterwards, two researchers, Breimer and Danny, have noticed that in the human AβPP nucleotide sequence, the portion encoding C99 is preceded immediately, contiguously, and in-frame by an AUG codon [156]. Just this observation would be a sufficient ground for the far-reaching speculations, but there was more to it. The AUG codon under discussion is positioned within the optimal translation initiation nucleotide context (known as the Kozak motif). Moreover, as if this were not enough, this particular AUG codon is singular in that of twenty methionine-encoding AUG codons in human AβPP mRNA, it is the only one embedded within the optimal translation initiation nucleotide context. Strikingly, not even the translation-initiating AUG encoding Met1 of human AβPP is situated within the optimal translation initiation nucleotide context. Such extraordinary localization of the AUG encoding Met671 of human AβPP has sweeping implications: (a) translation can potentially initiate from this position, and (b) the initiation of translation from the AUG under discussion would result in C99 (C100) and, subsequently, Aβ produced independently of AβPP.

### 8.2. Internal Initiation of Translation from the AUG Encoding Met671 of Human AβPP Cannot Be Ruled Out

Following their observation, Breimer and Danny reasoned that the unique and propitious localization of the AUG encoding Met671 of human AβPP may be not random but rather reflects the underlying physiological function [156]. They suggested that, in Alzheimer’s disease, translation of the intact AβPP mRNA initiates internally from the AUG encoding Met671 of AβPP and results in C99 generated independently of AβPP [156]. This proposition attracted significant interest and was eventually addressed experimentally by two research groups. The rationale for both attempts was that if translation indeed initiates internally from the AUG in question, manipulation of AβPP coding nucleotide sequence upstream from it would not interfere with the process. Accordingly, multiple frame-shifting mutations were introduced upstream from the AUG of interest in one study [157]. In another study, translational stop codon was inserted upstream of it [158]. Both studies reasoned that if translation initiates internally, the introduced mutations would not affect it. However, in both studies, the mutations completely stopped the production of C99 and Aβ, and, consequently, the internal initiation of translation was ruled out. This conclusion may be correct, but for the wrong reasons. The process posited by Breimer and Danny in [156] was proposed to occur in AD-affected human neurons in a disease-inducible manner. Both studies described above [157,158], however, were carried out in non-neuronal cells and definitely not under AD conditions. Breimer and Danny’s proposition [156], therefore, remains potentially valid and should be reevaluated in an adequate human neuronal cell-based model system.

### 8.3. Internal Initiation of Transcription Can Produce 5′-Truncated AβPP mRNA where the AUG Encoding Met671 Is the First Translation Initiation Codon

The unconventional internal initiation of translation within the intact human AβPP mRNA is, however, not the only way to utilize the AUG encoding Met 671 for the AβPP-independent production of Aβ. The identical result can be achieved in a conventional manner by generating suitably 5′-truncated AβPP mRNA where the AUG in question becomes the first, 5′-most, in-frame translation initiation codon. One way to generate such 5′-truncated AβPP mRNA is via the internal initiation of transcription within the AβPP gene. Such initiation of transcription should, obviously, occur upstream of the AUG under discussion and it would lead to the production of mRNA encoding C99 (or rather C100, see below) and, subsequently to the generation of C99 independently of AβPP. This process would require the production of a specialized transcription factor or co-factor expressed in the AD-affected neurons, presumably as a result of the ISR-mediated transcriptional/translational reprogramming.

### 8.4. Site-Specific Cleavage of AβPP mRNA Can Also Generate Suitably 5′-Truncated AβPP mRNA

Another way to generate a suitably 5′-truncated AβPP mRNA where the AUG normally encoding Met671 is the first translation initiation codon is to cleave the intact AβPP mRNA in a site-specific manner. The requirements for the position of the site of such cleavage are the same as for that of the site of the internal initiation of transcription discussed above: it should be positioned upstream of the AUG in question, with no other functional in-frame translation initiation codons in-between. The primary translation product of so truncated mRNA would be C100 (see below) produced independently of AβPP. The truncated mRNA would be cap-less; it would be, nevertheless, a functional translation template under the conditions of the ISR (sustained, as discussed above, by *i*Aβ produced independently of AβPP), which enable cap-independent translation. Such site-specific cleavage of the intact AβPP mRNA would depend on the de novo production of a specialized nuclease, expressed, presumably, within the framework of ISR-mediated transcriptional/translational reprogramming.

### 8.5. Unconventional Generation of 5′-Truncated Chimeric AβPP mRNA Encoding the C100 Fragment of AβPP

Apparently, the most plausible mechanism underlying the operation of the AβPP-independent *i*Aβ production pathway is unconventional. It is the generation of 5′-truncated chimeric AβPP mRNA encoding the C100 fragment where the first in-frame translation initiation codon is the AUG normally encoding Met671 of AβPP; it is “chimeric” because its 5′ untranslated region (5′UTR) includes a 3′-terminal segment of the antisense AβPP RNA. The plausibility of this mechanism is strongly supported empirically, and it provides a mechanistic explanation as to why the AβPP-independent *i*Aβ production and, consequently, AD occur in humans but not in mice and in the current transgenic animal AD models; it also instructs how to overcome this limitation and construct an adequate animal AD model. Due to its potential importance, this mechanism and its application to AβPP-independent *i*Aβ production in AD are further discussed in the following three sections below.

## 9. RNA-Dependent Amplification of Mammalian mRNA: General Principles

Mammalian RNA-dependent mRNA amplification, described in detail elsewhere [159,160,161,162,163,164,165,166,167,168], occurs physiologically in situations requiring the large-scale production of specific proteins, for example, in some types of terminal differentiation [159,160,161] or during deposition of extracellular matrix proteins [162]. This process may potentially occur in two stages. The first stage is a “chimeric” pathway, named so because it yields a chimeric mRNA where a portion of or the entire 5′UTR consists of the 3′-terminal segment of the antisense RNA strand. In this pathway, every conventionally transcribed mRNA serves repeatedly as a template; this process is thus linear. Subject to certain requirements, it can, upon completion, expand into the second stage that operates in a PCR-like manner where every newly transcribed amplified RNA molecule serves as a transcription template; this process is exponential. The second mRNA amplification stage, although very interesting, has no relevance to the present discussion subject, and only the chimeric mRNA amplification pathway is described below. The latter is of special interest because it is capable of generating 5′-truncated human AβPP mRNA encoding only the C100 fragment.

**Figure 8:**
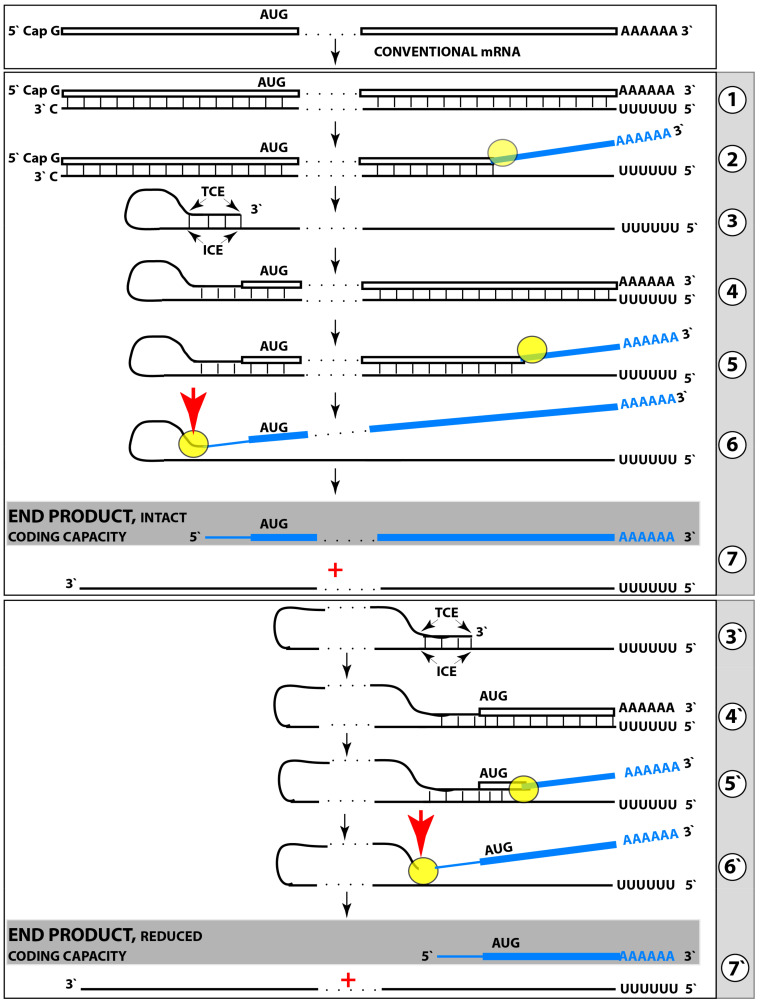
RNA-dependent amplification of mammalian mRNA amplification: principal stages. *Boxed line*: sense RNA. *Single line*: antisense RNA. “*AUG*”*:* codon for translation-initiating methionine. “*TCE*”: 3′-terminal complementary element of the antisense RNA; “*ICE*”: internal complementary element of the antisense RNA. *Yellow circle*: helicase complex; it also includes nucleotide-modifying activity. *Blue lines* (both single and boxed): RNA strands following their separation by the helicase/nucleotide-modifying complex. *Red arrows*: position of the cleavage of the chimeric RNA intermediate. (**Top panel**) Conventional, genome-transcribed mRNA, the “progenitor” in the mRNA amplification pathway. (**Middle panel**) Principal stages of the chimeric pathway of mammalian RNA-dependent mRNA amplification. Stage **1**: The antisense RNA is transcribed from the conventional mRNA progenitor by RdRp. Stage **2**: Sense and antisense strands are separated. The helicase complex mounts 3′-terminal poly(A) of the sense RNA and moves along it, modifying on average every fifth nucleotide along the way. Stage **3**: TCE–ICE-assisted folding of the antisense RNA into a self-priming configuration; Stage **4**: Extension of the self-primed antisense RNA. Its 3′ terminus is extended into the sense RNA, resulting in a hairpin-like structure. Stage **5**: Strand separation. The helicase complex mounts the 3′-terminal poly(A) of the sense strand and moves along it, separating it from the antisense strand. Simultaneously introduced nucleotide modifications presumably prevent the strands from re-annealing. Stage **6**: Upon reaching the single-stranded portion of the hairpin structure, the helicase complex cleaves the chimeric RNA intermediate; Stage **7**: 3′-truncated antisense RNA and chimeric mRNA end products of the chimeric mRNA amplification pathway. Note that in the above sequence, the ICE is positioned within a segment of the antisense RNA corresponding to the 5′UTR of the mRNA progenitor; in this scenario, the chimeric RNA end product contains the intact coding region of the conventional mRNA molecule. (**Bottom panel**) The ICE is positioned within a segment of the antisense RNA corresponding to the coding region of the mRNA progenitor. In this scenario, the amplified chimeric RNA end product contains a 5′-truncated coding region of the conventional mRNA progenitor. The translational outcome is defined by the position of the first translation initiation codon. If the first functional translation initiation codon is in-frame, translation would result in the C-terminal fragment of the conventionally encoded polypeptide. Stages **3′** through **7′** correspond to stages **3** through **7**.

The chimeric pathway of RNA-dependent amplification of mammalian mRNA is depicted schematically in the upper and middle panels of Figure 8. The amplification process begins with the generation of the antisense strand of a conventionally gene-transcribed mRNA molecule by the RNA-dependent RNA polymerase, RdRp. It initiates within the 3′-terminal poly(A) region of mRNA and results in a double-stranded RNA containing strands in both, sense and antisense, orientation. The double-stranded RNA structure is resolved by helicase activity, which commences at the 3′-terminal poly(A) and proceeds in the 5′ direction. When separated, conventionally produced mRNA can be repeatedly reutilized in the amplification process.

The newly synthesized antisense RNA, when separated from its mRNA template, undergoes folding and assumes a self-priming configuration. This requires the presence within the antisense RNA strand of two not only complementary (or sufficiently complementary) but also topologically compatible segments. One of this segments has to be strictly 3′-terminal [169] (referred to as the terminal complementary element, TCE) whereas another segment can be situated any place within the antisense RNA strand (referred to as the internal complementary element, ICE). The TCE–ICE may contain internal mismatches; the only requirement for them is to be capable of forming a stable double-stranded structure without 3′-terminal overhang. Upon formation of a self-priming structure, the 3′ terminus of the antisense strand is extended into RNA in the sense orientation; the position of the initiation of extension of the antisense into sense RNA constitutes the “chimeric junction”, i.e., the point of the conversion of RNA orientation. When the extension is completed, it produces a hairpin-like structure. The double-stranded portion of this structure is separated by a helicase complex invoked above. Upon reaching the single-stranded segment of the hairpin-like structure, the helicase cleaves the RNA molecule. The cleavage occurs either at the 3′ end of the hairpin loop or at one of the TCE–ICE mismatches.

One of the two resulting end products of the chimeric mRNA amplification pathway is the 3′-truncated antisense RNA. It misses either the entire TCE (if the cleavage occurs at the 3′ end of the hairpin loop) or a portion of it (if the cleavage occurs at the TCE–ICE mismatch). In the chimeric mRNA amplification pathway, the 3′-truncated antisense RNA end product is capable of limited re-use if the cleavage occurs at the TCE–ICE mismatch and leaves a substantial portion of the TCE intact (see below). However, if the amplification process is expanded and continues into the second stage, its fate is interesting and exciting: it gets polyadenylated at the 3′ end in conjunction with the cleavage, and serves as the initial template in the PCR stage of amplification {it has poly(A) at the 3′ end and poly(U) transcribed from the 3′-terminal poly(A) of mRNA at its 5′ end; its transcript, generated by RdRp, would likewise possess 3′-terminal poly(A) and 5′-terminal poly(U)} [160,161]. Another, functional (i.e., protein-encoding), end product of the chimeric amplification pathway is chimeric mRNA. It consists of the 5′-truncated mRNA and a covalently attached segment of the antisense RNA, actually its cleaved-off TCE (if the cleavage took place at the 5′ end of the TCE) or a portion thereof (the latter if the cleavage occurred at the TCE–ICE mismatch). The anticipated uncapped nature of the amplified chimeric mRNA end product is consistent and functionally compatible with the preferential cap-independent type of translation under the ISR conditions. In the reported cases of mammalian RNA-dependent mRNA amplification, those of globin and laminin mRNAs [159,160,161,162], the folding of the antisense RNA occurs within its segment corresponding to a portion of the 5′UTR of mRNA. This is the scenario depicted in the middle panel of Figure 8. In such cases, the resulting chimeric mRNA retains the complete coding capacity of the conventional mRNA progenitor, and upon translation yields the polypeptide identical to the translational product of the conventional mRNA. This, however, is not always the case.

## 10. Asymmetric RNA-Dependent Mammalian mRNA Amplification: Amplified mRNA Encodes Only CTF of the Conventionally Produced Polypeptide

The 3′-terminal TCE is, as reflected in its designation, always 3′-terminal. This is a strict requirement crucial for its potential to prime transcription of RNA. In contrast, the ICE can be any place within the antisense RNA. If the ICE occurs within a portion of the antisense RNA corresponding to the 5′UTR of conventional mRNA, the amplified mRNA would retain the complete coding capacity of the mRNA progenitor, but this is only one of many possibilities (for detailed discussion of the subject, see [164]). For example, the amplified chimeric RNA may encode a CTF of the original protein or even a polypeptide non-contiguously encoded in the genome [164]. What scenario will play out depends on two factors. One is the position of the ICE within the antisense RNA. It defines the site of the initiation of transcription and the extent of the 5′ truncation of the amplified mRNA. Another factor is the position of the first functional translation initiation codon within the amplified chimeric RNA.

The present section considers a scenario that results in the production of CTF of a conventional protein. This scenario is of special interest due to its relevance to the potential of generating C99 (a CTF) independently of AβPP. In this scenario, diagrammatically illustrated in the bottom panel of Figure 8, the ICE is positioned within a segment of the antisense RNA corresponding to the coding region of conventionally produced mRNA. Consequently, self-primed extension of the 3′ terminus of the antisense RNA would generate only the 3′-terminal segment of conventional mRNA containing only a 3′ portion of its coding region. Subsequent strand separation and cleavage would produce a chimeric mRNA truncated within its coding region. The translational outcome of this scenario would be defined by the position of the first functional translation initiation codon (AUG or any other initiation-competent codon). If it occurs within the remaining portion of the coding region and if it were in-frame, translation initiated at this position would generate a CTF of conventionally produced polypeptide. Thus, this type of RNA-dependent mRNA amplification would be asymmetric: the amplified RNA would contain only the 3′-terminal portion of the coding region of mRNA progenitor and would yield, upon translation, only a CTF of the conventional polypeptide.

## 11. Human AβPP mRNA Is RdRp-Compatible Template Eligible for Asymmetric Amplification Yielding 5′-Truncated, C99-Encoding mRNA

The asymmetric pathway of chimeric mRNA amplification appears to be, in principle, capable of generating C99 (C100) independently of AβPP, provided that in the resulting 5′-truncated chimeric mRNA the first functional translation initiation codon were the AUG encoding Met671 of AβPP. There are, however, many barriers on the way. One is the distances (in nucleotides) involved in this process. The AUG encoding Met671 is situated over 2000 nucleotides downstream from the 5′ terminus of human AβPP mRNA. Provided that suitable TCE and ICE do occur in the antisense RNA, the location of the ICE would have to be 3′ from but in the vicinity of the complement of the AUG in question and thus separated from the TCE also by about 2000 nucleotides. Given the highly complex nature of RNA folding, especially over long distances, the question is whether the TCE and ICE (if they are present within the antisense AβPP RNA to begin with) would be topologically compatible, i.e., would they find each other in the folded antisense RNA configuration? In other words, the question is whether human AβPP mRNA is the eligible template for RNA-dependent mRNA amplification. Its eligibility can be assessed in the following general approach, applicable to any mammalian mRNA species; actually, any RNA species containing 3′-terminal poly(A).

### 11.1. Evaluation of the Eligibility of an mRNA Species for the Chimeric mRNA Amplification Process: General Approach

To assess the eligibility of any mRNA species for RNA-dependent mRNA amplification, the mRNA of interest is first transcribed by reverse transcriptase (RT) starting at the 3′-terminal poly(A) segment. This produces its antisense strand, cDNA. The mRNA component is then removed by RNase H, usually present in preparations of RT (unless eliminated genetically). At this point, we have the antisense strand, separated from its mRNA template, in the presence of an enzyme (RT) capable to extend it if it forms a suitable self-primed structure, a situation equivalent to that depicted in the initial stages of Figure 8. If the resulting cDNA is the complete transcript of mRNA, if it contains the TCE and ICE, if the TCE and ICE (provided they are present in the cDNA) are topologically compatible, the cDNA would fold into a self-priming configuration. RT would then extend the 3′ end of cDNA into the sense strand. The nucleotide-sequencing analysis would determine whether the extension occurred, thus defining whether or not the topologically compatible TCE and ICE are present in the antisense transcript. If affirmative, this would establish the eligibility of the mRNA species of interest for RNA-dependent mRNA amplification, and the junction between the antisense and sense portions of the resulting product would determine the site of the initiation of self-primed extension, define the 5′ end of the ICE and enable the identification of both complementary elements.

### 11.2. Human AβPP mRNA Is the Eligible Template for Asymmetric Amplification Resulting in mRNA Encoding C100

The evaluation described above was, in fact, carried out, albeit inadvertently, for human AβPP mRNA. As was mentioned above, in 1987, three research groups cloned and sequenced human AβPP cDNA. Shortly after these studies were published, one group reported cloning and sequencing of much larger human AβPP cDNA that was substantially extended at its 3′ terminus [170]. This study speculated that the 3′-extended AβPP cDNA was templated by the corresponding 5′-extended AβPP mRNA, and that this 5′-extended mRNA, in turn, originated by the initiation of transcription at a site upstream from the transcription start site (TSS) defined by previously sequenced AβPP cDNAs [153,154,155]. At the time [170] was published, the genomic sequence upstream from human AβPP was not yet known. Soon afterwards, however, it was determined [171], and it became apparent that the reported 3′ extension of human AβPP cDNA did not correspond to and therefore could not have originated from a genomic transcript. As a result, the group that detected the 3′-extended human AβPP cDNA declared their finding an artifact and published a correction to this effect [170] (correction).

However, the careful analysis of the 3′-extended human AβPP cDNA in question showed that the extension is actually a segment of a human AβPP sense strand [172,173,174]. This result leaves little doubt regarding the origin of the extended segment. It is clear that it was produced during the preparation of cDNA by its self-primed extension, which occurred just as described in the preceding subsection. The analysis of the antisense–sense junction showed that the extension initiated about 2000 nucleotides from the 3′ end of the cDNA. It also defined the 5′ end of the ICE and thus allowed the identification of the TCE and ICE sequences [172,173,174]. These results thus established that human AβPP mRNA is eligible for RNA-dependent mRNA amplification. Moreover, the results affirmed that amplification of human AβPP would occur asymmetrically. The next key question is where translation of the amplified chimeric RNA would initiate. As became clear from the sequence analysis, the first translation initiation codon within the extended sense-orientation portion of cDNA is actually the ATG encoding Met671 of human AβPP [172,173,174].

**Figure 9:**
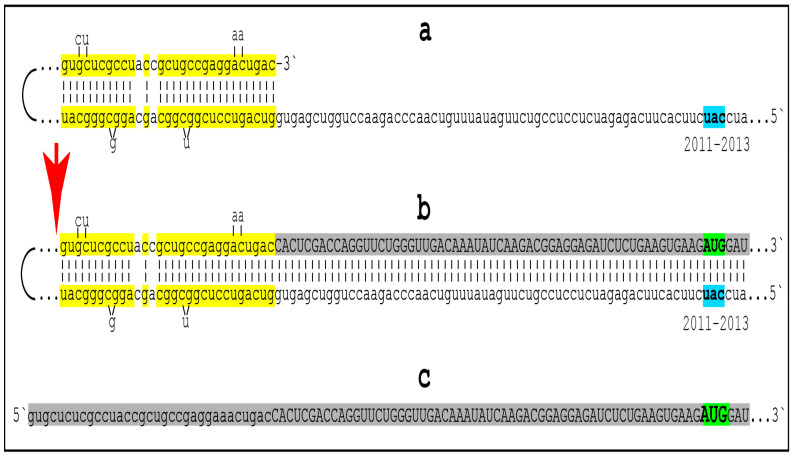
Human AβPP mRNA is the eligible template for asymmetric amplification resulting in mRNA encoding C100. *Lowercase letters*: nucleotide sequence of the antisense RNA. *Uppercase letters*: nucleotide sequence of the sense RNA. Highlighted *in yellow*: the TCE (top) and the ICE (bottom) elements of the human antisense AβPP RNA. *“2011–2013”*: nucleotide positions (counted from the 3′ terminus of the antisense AβPP RNA) of the “uac” (highlighted *in blue*), the complement of the “AUG” (highlighted *in green*) encoding Met671 in the human AβPP mRNA. Panels (**a**–**c**) correspond to stages 3′, 4′, and 6′ of Figure 8. (**a**) TCE–ICE-assisted folding of the human AβPP antisense RNA into the self-priming conformation. (**b**) Extension of the self-primed AβPP antisense RNA into the sense RNA (highlighted *in gray*). *Red arrow*: cleavage of chimeric RNA intermediate following separation of sense and antisense RNA. Note that the cleavage is shown at the 3′ end of the single-stranded loop portion of the hairpin structure; it may also take place at one of the mismatches within the TCE–ICE. (**c**) Chimeric RNA end product of RNA-dependent amplification of human AβPP mRNA (highlighted *in gray*). It contains the antisense portion extended into the 5′-truncated coding region of human AβPP mRNA (note that the antisense–sense junction can shift if the cleavage occurs at one of the TCE–ICE mismatches; this aspect is addressed in Section 14 below). In the chimeric RMA end product, the first translation initiation codon is the in-frame AUG (highlighted *in green*) that encodes Met671 of human AβPP; when translated, the chimeric mRNA end product would produce C100 independently of AβPP.

The projected folding into self-priming configuration and the extension of human antisense AβPP RNA into the sense strand encoding the C100 fragment of AβPP is presented in Figure 9 (panels “a,” “b,” and “c” correspond to stages 3′, 4′, and 6′ in Figure 8). As shown, human antisense AβPP RNA possesses the TCE and ICE, which are separated by over 2000 nucleotides. They are, nevertheless, topologically compatible, i.e., mutually accessible in the folded RNA structure. If and when RdRp is available, the TCE, acting as a primer, is extended into the sense AβPP RNA. Upon completion of the extension process, complementary strands are separated by the helicase complex and the cleavage takes place either at one of the TCE–ICE mismatches or at the 5′ terminus of the TCE (only the latter is shown in the Figure). The process is asymmetric. It generates chimeric mRNA whose antisense portion consists either of the TCE or of the portion thereof (if cleavage occurs at one of the TCE–ICE mismatches). Its sense portion consists of the 5′-truncated coding region and the 3′UTR. In terms of the translation outcome, its most important attribute is the presence of the in-frame translation initiation codon. It is located 58 nucleotides from the chimeric junction and it is the AUG encoding Met 671 of AβPP. Upon translation of this RNA, C99 (C100) and, subsequently, Aβ would be produced independently of AβPP.

## 12. Testing for the Occurrence of the AβPP-Independent Production of *i*Aβ in the Human Neuronal Cell-Based AD Model

The production of C99 and, consequently, of Aβ independently of AβPP is the quintessential and cardinal requirement within the framework of the ACH2.0. Obtaining a proof for this phenomenon would firmly establish the new AD paradigm. Auspiciously, making this determination appears feasible. When Breimer and Danny [156] suggested that the AUG encoding Met671 of AβPP could be used in the initiation of translation, obtaining such proof seemed unattainable. Indeed, according to the state of art at that time, it was assumed that, following the initiation of translation at the AUG in question, the initiating methionine would be removed co-translationally, as commonly happens, by the N-terminal methionine aminopeptidases 1/2 (MAP1/2). The resulting primary product, therefore, would be C99 that could be cleaved by gamma-secretase to generate Aβ. Thus, in the Breimer and Danny’s narrative [156], the products of translation initiated at the AUG in question (independently of AβPP) would be identical in every way to and indistinguishable from the corresponding products of the AβPP proteolysis. However, the ensuing investigations into the processing of the N-terminus of the newly synthesized polypeptides have proven this presumption incorrect.

### 12.1. Initiation of Translation at the AUG Encoding Met671 of AβPP Results Not in C99 but in the C100 Fragment: The Initiating Methionine Is Not Removed Co-Translationally

Translation of the overwhelming majority of cellular proteins is initiated with methionine. The initiating methionine is usually removed co-translationally by the MAP1 or MAP2, and the resulting primary translation product starts at the N-end with a residue that follows the translation-initiating Met. This, however, is not always the case [175,176,177,178,179,180]. For the translation-initiating methionine to be cleaved co-translationally, it, and the residue that follows it, should be accommodated within the active site of MAP1/MAP2. This is, strictly speaking, a topological requirement and it cannot always be satisfied. With the size of the initiating methionine constant, the dimensions of the pair are defined by the size of its second residue. The size of an amino acid residue is determined by the radius of gyration of its side chain (RG). The RG is zero in glycine (no side chain), 0.77 Angstrom in alanine, 1.08 in serine, 1.22 in cysteine, 1.24 in threonine, 1.25 in proline, and 1.25 in valine. When the translation-initiating methionine is followed by any of these seven residues, the pair fits within the MAP1/MAP2 active site and the cleavage occurs co-translationally. When the translation-initiating methionine is followed by any other residue (all of them larger than valine), the pair cannot be accommodated within the MAP1/MAP2 active site, co-translational cleavage does not occur, and the primary translation product retains the initiating methionine [175,176,177,178,179,180].

Met671 of human AβPP is followed by the aspartate (RG 1.43 Angstrom). The Met/Asp combination does not fit within the MAP1/MAP2 active site. Therefore, if translation initiates with the methionine normally in position 671 of AβPP (regardless of the mechanism underlying such translation initiation), the initiating Met is not removed co-translationally and the primary translation product is the C100 (Met-C99) fragment of AβPP. This is not a unique situation; in cases like this, when the initiating Met is retained in the primary translation product, it is usually removed by one of the numerous aminopeptidases with a broad specificity [180]. Thus, C100 is eventually converted into C99. If C100 were cleaved by gamma-secretase prior to the removal of its N-terminal methionine, this would result in Met-Aβ. In such a case, Met-Aβ would be eventually converted into Aβ by the same mechanism that converts C100 into C99. Importantly, the removal of the N-terminal translation-initiating methionine by an aminopeptidase other than MAP1/MAP2 unvaryingly occurs post-translationally.

### 12.2. Validation of the AβPP-Independent Production of C100 and Met-Aβ

Since the removal of the N-terminal methionine from C100 and Met-Aβ would always occur post-translationally, their pools should be present in the AD-affected human neuronal cells. The sizes of these pools would reflect the rate of the removal of the translation-initiating methionine, and, in the case of Met-Aβ, the relative rates of the aminopeptidase versus gamma-secretase cleavages, but these pools should exist in live AD-affected neurons. The occurrence of such pools would report on and validate the operation of the AβPP-independent *i*Aβ production pathway regardless of the nature of an underlying mechanism. The human neuronal cell-based AD model is suitable for testing the validity of the production of Aβ independently of AβPP. It should be commented at this point that the C100 and Met-Aβ pools would not be present in postmortem samples. This is because in dying cells, synthetic processes cease well in advance of proteolytic cleavages. Accordingly, in the absence of the influx of C100 and Met-Aβ, the remaining initiating methionine-containing molecules would be rapidly and completely converted into C99 and Aβ. An alternative, albeit more complicated, approach to validate the occurrence of the AβPP-independent production of C100 initiating at the AUG normally encoding Met671 of AβPP is described in the following section.

## 13. Elucidation of Molecular Nature of the AβPP-Independent *i*Aβ Production Mechanism in Human Neuronal Cell-Based AD Model

The occurrence of the initiation of translation from the AUG encoding Met671 of AβPP can also be assessed utilizing the same procedures as those employed previously [157,158] but in the appropriate model system, a human neuronal cell-based AD model. More specifically, a frame-shifting mutation or a translational stop-codon mutation can be edited in the endogenous AβPP gene upstream from the AUG encoding Met671. If the initiation of translation from the AUG in question does not occur, no Aβ would be produced endogenously in this model system. If it does, neither mutation would interfere with it. The criterion for the occurrence of the endogenous Aβ generation would be the presence of C100 and, possibly, Met-Aβ (more precisely, the presence of the N-terminal methionine in these molecules).

In another approach, in addition to the mutations upstream from the potentially translation-initiating AUG in the endogenous AβPP gene, one of the FAD mutation can be introduced within the Aβ-coding segment of the endogenous AβPP gene or, alternatively, within the Aβ-coding segment of a transgene. In either case, Aβ produced endogenously would be distinguishable from exogenously expressed Aβ, and the presence of the former would validate the occurrence of the endogenous initiation of translation at the AUG encoding Met671 of AβPP independently of the appearance and detection of the N-terminal Met in C100 or Met-Aβ. This approach can complement or substitute the detection of C100 or Met-Aβ as the validation test and could be useful if C100/Met-Aβ pools are too small for their unambiguous detection. The modification of the endogenous AβPP gene in this approach should be minimal in order not to interfere with the folding of the antisense AβPP RNA (e.g., the translational stop codon could be introduced with the replacement of a single nucleotide).

The relevant studies discussed above [157,158] attempted similar approaches for determination of the occurrence of the internal initiation of translation within the intact AβPP mRNA. However, the experiments described in [157,158] and in the present section above address only the possible initiation of translation from the AUG encoding Met671 of human AβPP. They cannot distinguish between the mechanisms underlying this phenomenon. If and when the occurrence of the AβPP-independent *i*Aβ production in the human neuronal cell-based AD model is verified as discussed above, the subject of the nature of the mechanism underlying the operation of this pathway can be considered. Thus, the assignment of the origin of such phenomenon to the internal initiation of translation within the intact human AβPP mRNA would require evaluation of the operational occurrence of the IRES, which can be addressed using standard analytical approaches [181,182,183,184,185,186].

Whether the internal initiation of endogenous transcription within human AβPP gene or the site-specific cleavage of the intact human endogenously produced AβPP mRNA do take place, both occurring upstream of the AUG encoding Met671 of AβPP, could be suggested by the observation of the appropriately 5′-truncated AβPP mRNA where the first translation initiation codon is the AUG in question. If such RNA were observed, the presence of the cap structure at its 5′ terminus would indicate the occurrence of the internal initiation of transcription. If the 5′-terminal cap structure were not present, this would be consistent with the site-specific cleavage of the intact AβPP mRNA (for the approaches to detect the operation of the chimeric RNA-dependent human AβPP mRNA amplification pathway, see Section 14 below).

The procedures to evaluate the remaining and, arguably, the most plausible option, namely, that the human AβPP-independent production of IAβ is enabled by the asymmetric RNA-dependent amplification of human AβPP mRNA in the chimeric pathway, are analyzed in detail in the following section.

## 14. Validation of the Occurrence of Asymmetric AβPP mRNA Amplification: Search for the Chimeric RNA Intermediate

### 14.1. Why the Chimeric RNA Intermediate (Rather than Chimeric mRNA End Product)?

To assess the possibility that the AβPP-independent *i*Aβ production pathway is powered by the asymmetric RNA-dependent amplification of human AβPP mRNA in the chimeric pathway, it is sufficient to test for the presence of the chimeric mRNA end product. As shown in stage 7′ of Figure 9, the end product of human AβPP mRNA amplification would contain severely 5′-truncated coding region and, at its 5′-terminus, as a part of the 5′UTR, a 3′-terminal segment of the AβPP antisense RNA, more precisely its TCE element or a part thereof (if the cleavage occurs at one of the TCE–ICE mismatches). Due to the nature of the amplification process, where every conventionally genome-transcribed AβPP mRNA can be repeatedly utilized as a template (a situation analogous to a massive gene amplification), the chimeric mRNA end product should be highly ubiquitous and thus easy to detect. This, however, is not the case. This is because when the extension of the self-primed antisense RNA is completed, the helicase complex mounts the newly synthesized 3′ poly(A) and proceeds along this strand, separating it from its template (sages 5, 5′ and 6, 6′ in Figure 9). While separating this RNA strand, the helicase activity also modifies on average its every fifth nucleotide [160,161]. Ostensibly, the function of these modifications is to prevent the re-annealing of just-separated strands: the nature of these modifications is such that they interfere with nucleic acid hybridization [160,161]. Consequently, they also interfere with cDNA synthesis and cDNA-based sequencing. Therefore, the chimeric mRNA end product, although ubiquitous, cannot be used as a reporter of the RNA-dependent mRNA amplification process [160].

Therefore, the role of a reporter falls to its immediate precursor, the chimeric intermediate of the mRNA amplification. The region of interest that defines the chimeric nature of the molecule, its “identifier,” is the region around and including the junction of sense and antisense segments, the “chimeric junction.” The chimeric junction is created concurrently with the commencement of the extension of the self-primed antisense RNA. At this point, it is not modified. It remains unmodified for the duration of the extension (stages 4, 4′ in Figure 9) and for the time period it takes the helicase complex to traverse the distance between the 3′-terminal poly(A) and the vicinity of the chimeric junction (stages 5, 5′ in Figure 9). This means that there is a defined duration, roughly between the commencement of the extension of the self-primed antisense RNA and the cleavage generating the chimeric RNA end product, when the chimeric junction region is not yet modified and is therefore detectable by the conventional means. If detected, the occurrence of the chimeric junction sequences would report on the occurrence of the RNA-dependent amplification of human AβPP mRNA. Such an approach was indeed validated in the detection of chimeric junctions generated during RNA-dependent amplification of globin and laminin mRNAs [160,161,162].

### 14.2. Potential Human AβPP mRNA Amplification-Generated Chimeric Junctions

As discussed above, each conventionally genome-transcribed mRNA molecule can be repeatedly utilized in cycles of amplification. Each cycle produces one antisense RNA molecule, which in turn might produce several distinctly different chimeric RNA intermediates. If the TCE and ICE elements are present within this molecule and are fully complementary, each antisense RNA transcript would produce only one chimeric RNA intermediate (the full-size antisense RNA extended into sense RNA) and, correspondingly, only one chimeric mRNA end product: the helicase complex would advance to the 5′ end of the TCE and cleave it off, as shown in stages 6, 6′ in Figure 9. In such a case, only one chimeric RNA intermediate molecule would be generated, with chimeric junction occurring at the 3′ end of the TCE element. If, however, the TCE–ICE combinations contain mismatches (and in all described cases of RNA-dependent mRNA amplification they do), the outcome would be different. In such a case, the helicase would cleave at the first (3′-most) mismatch, liberating the chimeric RNA end product with only a 3′-terminal portion of the TCE at its 5′ end. If the self-primed antisense RNA structure remains stable (despite its shortened TCE), it would be capable of another extension. In the resulting chimeric RNA intermediate, however, the chimeric junction would be shifted because the TCE is shorter (missing it cleaved-off 3′-terminal portion). This phenomenon is referred to as the “chimeric junction shift” and was, in fact, observed empirically [160,161]. If more than one mismatch is present in a particular TCE–ICE pair, more than one chimeric junction shift may occur and more than one chimeric RNA intermediate (and, subsequently, more than one chimeric RNA end product) would be generated from one antisense RNA molecule.

### 14.3. Differentiating between Mechanistic Possibilities

The putative end product of the RNA-dependent human AβPP mRNA amplification is a cap-less RNA molecule, which cannot be translated under the regular conditions. It would be, however, preferentially translated under the ISR conditions. This circumstance opens up an alternative mechanistic explanation of the AβPP-independent *i*Aβ production in the AD-affected neurons. Indeed, it can be speculated that the RNA-dependent amplification of human AβPP mRNA does occur constitutively in healthy AD-unaffected neurons but the resulting chimeric mRNA product remains untranslated, “silent.” In such a case, the elicitation of the ISR, rather than activating the RNA-dependent mRNA amplification, enables translation of the otherwise “silent” uncapped C100-encoding chimeric mRNA. The assay described above would allow distinguishing between the two possibilities. If chimeric intermediates of AβPP mRNA amplification were found in human neuronal cell-based model system only when the ISR is elicited, this would indicate the inducible nature of the amplification process. If, on the other hand, the intermediates were detected also prior to the ISR elicitation, this would suggest the constitutive operation of the AβPP mRNA amplification mechanism in the human neuronal cells.

### 14.4. Putative Nucleotide Sequences of Human AβPP mRNA Amplification-Generated Chimeric Junctions to Search for in Human Neuronal Cell-Based AD Model

**Figure 10:**
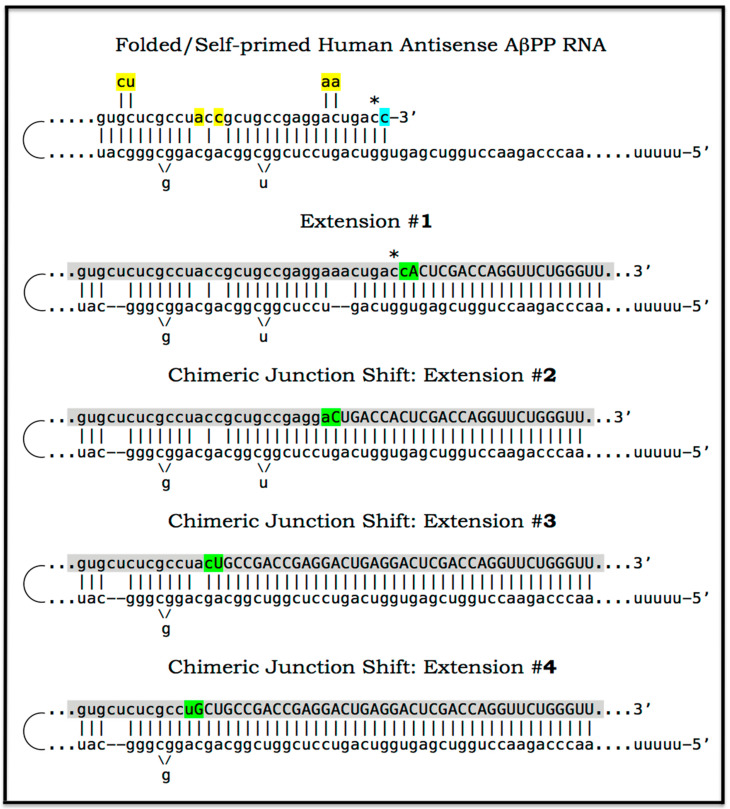
Chimeric junction shifts can generate multiple distinct chimeric RNA intermediates: the nucleotide sequences to look for. *Lowercase letters*: nucleotide sequence of the antisense RNA. *Uppercase letters*: nucleotide sequence of the sense RNA. Highlighted *in yellow*: mismatches within the TCE–ICE complex. Highlighted *in green*: chimeric junctions, the sites of the initiation of the extension of the self-primed antisense RNA into the sense RNA strand. *Asterisk*: the nucleotide position on the antisense RNA corresponding to the TSS of human AβPP mRNA (position (−)149 upstream from the “A” of the translation-initiating AUG codon). Highlighted *in blue*: “C” transcribed from the 5′-terminal cap “G” of AβPP mRNA (not encoded in the genome; addressed in Section 20 below). Highlighted *in gray*: regions containing chimeric junctions, the “identifier” nucleotide sequences to look for. “Extension #1” of the self-primed AβPP antisense RNA produces chimeric RNA intermediate containing the full-size TCE. If, at the strand separation stage, the cleavage occurs at the first (3′-most) mismatch and the self-priming antisense RNA structure (now with a shorter TCE) remains stable, it would be capable of another extension. In the resulting chimeric RNA intermediate, however, the chimeric junction would be shifted because the TCE is shorter (missing its cleaved-off 3′-terminal portion). If more than one mismatch is present, more than one chimeric junction shift may occur (“Extension #2,” etc.) and more than one chimeric RNA intermediate (and, subsequently, more than one chimeric RNA end product) would be generated from one antisense RNA molecule. In cases of RNA-dependent human AβPP mRNA amplification, there is potential for the generation of three or four different chimeric RNA intermediates, and, accordingly, for the detection of three or four distinct chimeric junctions. The nucleotide sequences to search for are highlighted in gray.

In cases of RNA-dependent human AβPP mRNA amplification, there is potential for the generation of three or four different chimeric RNA intermediates, and, accordingly, for the detection of three or four distinct chimeric junctions. One arises by the initial extension of the self-primed human antisense AβPP RNA whereas others are generated via the chimeric junction shift in subsequent extension events. The mechanics of this process as well as the sequences of the putative chimeric RNA intermediates are presented in Figure 10. The detection of any of the chimeric junctions would constitute a proof of the occurrence of the chimeric RNA-dependent amplification of human AβPP mRNA.

As was mentioned above, in the Authors’ opinion and for the reasons discussed above, the asymmetric RNA-dependent amplification of human AβPP mRNA in AD-affected neuronal cells appears to be the most plausible mechanism powering the AβPP-independent *i*Aβ production pathway. For the purposes of the further discussion, it is presumed that this is the case. Even if this were not the case, the following discussion would illuminate the general principles of construction of an adequate transgenic animal model of AD, which are applicable to other potential mechanisms underlying the production of *i*Aβ in the AβPP-independent manner.

## 15. Generation of an Adequate Transgenic Animal Model of AD, Part I: Elicitation of the ISR in Neuronal Cells

### 15.1. Utilization of the Current Transgenic Animal AD Models

In light of the above discussion, the generation of an adequate transgenic animal model of AD consists of two parts. The first part is the elicitation of the integrated stress response in the neuronal cells. A practical approach to this is, apparently, the utilization of the current transgenic animal AD models. In these models, exogenously produced *i*Aβ accumulates via its importation from the extracellular pool and through its intraneuronal retention following the gamma cleavage of C99 on the intracellular membranes. Moreover, as discussed above, it is apparent that in these models, the levels of exogenous *i*Aβ have crossed the T1 threshold and triggered the elicitation of the ISR. This is because under ISR conditions, the de novo protein synthesis is reprogrammed and severely suppressed. Since neuronal plasticity, long-term memory and learning capacity all depend on de novo protein synthesis, they are all impaired under the ISR. As discussed above, inhibition of the ISR by the small-molecule inhibitor ISRIB abrogates this impairment, and the prevention of the elicitation of the ISR by genetic and pharmacological means precludes this impairment in the current transgenic AD models [37,38,39,40,41,42,43,44,45]. It follows that the first requirement for the successful generation of an adequate transgenic model of AD is already fulfilled in the current models.

### 15.2. Elicitation of the ISR in Neuronal Cells via Exogenous Overexpression of Intraneuronally Retained iAβ42

The current transgenic animal models of AD are ineffective in the accumulation of *i*Aβ. Although in these models AβPP is massively overproduced, only a small fraction of the resulting Aβ ends up inside the neuron as *i*Aβ. Indeed, only a small fraction of secreted Aβ is imported back inside the cell and only a small fraction of AβPP is processed (including the gamma-cleavage) on the intracellular membranes thus yielding *i*Aβ. The rate of accumulation of AβPP-derived *i*Aβ can potentially be greatly increased if the bulk or the entire production output of exogenous Aβ is retained intraneuronally. This can be achieved by employing transgenes encoding only Aβ. In these models, the AUG normally encoding Met671 of AβPP would be utilized as the translation initiation codon. Since it is situated within the optimal initiation context, translation would be efficient. As discussed above, the primary translation product would be not Aβ, but Met-Aβ. The translation-initiating methionine, however, would be removed post-translationally by one of the cellular aminopeptidases with broad specificity; this will generate Aβ. Since it is lacking the transmembrane domain, this Aβ would be retained intraneuronally as *i*Aβ. Expressing exogenously *i*Aβ42 would be more efficient in the elicitation of the ISR than utilizing other isoforms of Aβ. This is because, due to its propensity to aggregate, *i*Aβ42 is more cytotoxic than other Aβ isoforms and would lower the T1 threshold for the activation of eIF2α kinases and the consequent elicitation of the ISR (discussed in detail in [4]).

### 15.3. iAβ-Independent Elicitation of the ISR in Neuronal Cells

In conventional AD, the elicitation of the integrated stress response is triggered by the accumulation of AβPP-derived *i*Aβ over the T1 threshold. However, within the framework of the ACH2.0, elicitation of the ISR in neuronal cells by any means, including stressors other than *i*Aβ, should be sufficient to provide the “missing” components of the mechanism enabling the AβPP-independent *i*Aβ production pathway and to activate its operation. Such *i*Aβ-independent elicitation of the ISR apparently underlie the cases of unconventional AD, such as those that are caused by traumatic brain injury, chronic encephalopathy, chronic inflammation, and viral and bacterial infection. The choice of a stressor in this approach would be largely defined by which one of the four eIF2α kinases is targeted for the activation. For example, for the activation of the HRI kinase, mitochondrial dysfunction could be triggered, whereas viral infection could be employed for the activation of the PKR kinase; the activation of one or more of the eIF2α kinases would be followed by the elicitation of the ISR. When the AβPP-independent *i*Aβ production pathway is activated in an AD model, it must be self-sustainable in order to drive the AD pathology. Whereas the AβPP-independent *i*Aβ production pathway triggered by the accumulation of AβPP-derived *i*Aβ over the T1 threshold is always self-sustainable (discussed above), this is not necessarily the case when the activation of this pathway is triggered by a stressor other than *i*Aβ; this issue is addressed in the following section.

## 16. Generation of an Adequate Transgenic Model of AD, Part II: Securing Self-Sustainable AβPP-Independent Production of *i*Aβ

The second part of the construction of an adequate transgenic animal model of AD is securing self-sustainable AβPP-independent production of *i*Aβ. The operation of this pathway seems to be essential for the occurrence of the disease [1,2,3,4,5,6]. Without it, the levels of *i*Aβ required to drive the AD pathology apparently cannot be achieved. In conventional AD, when the AβPP-independent *i*Aβ generation pathway is activated, it is immediately self-sustainable. This is because the initial stressor eliciting the ISR, in this case *i*Aβ, is already over the T1 threshold, and its production independently of AβPP assures that it stays so and, in addition to driving the AD pathology, propagates is own production. This would also be the case in transgenic animal models of the disease where the elicitation of the ISR is achieved through the accumulation of exogenous *i*Aβ over the T1 threshold.

If the ISR is elicited in neuronal cells by a stressor other than *i*Aβ, and, in turn, initiates the generation of *i*Aβ independently of AβPP, the activated AβPP-independent *i*Aβ production pathway would not be immediately self-sustainable. Indeed, if the initial ISR-eliciting stressor is withdrawn, operation of the AβPP-independent *i*Aβ production pathway would cease. For it to become self-sustainable, the ISR should be in effect for a sufficient duration to allow *i*Aβ produced independently of AβPP to accumulate over the T1 threshold. Repeated elicitations of the ISR for shorter durations would also achieve this by increasing the baseline levels of *i*Aβ (produced independently of AβPP) stepwise until they reach and cross the T1 threshold. However it is done in a perspective transgenic AD model where the ISR is initially elicited independently of *i*Aβ, for the model to be adequate, it must be ascertained that the ISR-activated AβPP-independent *i*Aβ production pathway attains self-sustainability. In unconventional cases of AD, both approaches mentioned above are, apparently, utilized: in chronic encephalopathy it is the repeated trauma (i.e., the repeated elicitation of the ISR and a stepwise increase in the levels of *i*Aβ produced independently of AβPP), whereas in chronic inflammation and prolonged viral and bacterial infections, it is a sufficient duration of the ISR elicitation, which ensures the accumulation of *i*Aβ (produced in the AβPP-independent pathway) to levels exceeding the T1 threshold.

The above considerations are relevant and applicable only if the integrated stress response *does* activate the AβPP-independent generation of *i*Aβ. This, however, *does not* happen in the current transgenic animal models of AD. Why this is so and how to overcome this deficiency is addressed in the following sections.

## 17. Mice Possess and Utilize Physiologically the RNA-Dependent mRNA Amplification Pathway

One way to explain why the AβPP-independent *i*Aβ production pathway is inoperative in mice is to assume that this species does not possess the machinery required to enable the pathway’s activity. In more precise terms, the assumption would be that mice lack the functional RNA-dependent mRNA amplification-competent RdRp. Such an assumption, however, would be incorrect. RNA-dependent mRNA amplification-competent RdRp was shown to be present and vigorously operating in mice physiologically. Two such instances have been described in detail. One is RNA-dependent amplification of alpha and beta globin mRNAs in differentiating erythroid cells [159,160,161]. The power of this process in terms of the production of mRNA is considerable: at the height of the erythroid differentiation the amount of globin mRNA produced in the RNA-dependent amplification pathway is about 1500 fold greater than the amount of globin mRNA generated conventionally during the same period in the same cells [160,161]. Another example is RNA-dependent amplification of mRNA species encoding alpha1, beta1, and gamma1 laminin in cells producing and secreting extraordinary amounts of this extracellular matrix component [162].

As in both examples above, the RNA-dependent mRNA amplification pathway appears to operate in circumstances requiring rapid production of immense quantities of particular polypeptides; thus, in erythroid differentiation cells are literally filled with hemoglobin (resulting in reticulocytes and, eventually, erythrocytes) within 48–72 h. Arguably, the extracellular matrix proteins are produced in even greater quantities within comparable time periods. In both examples and, probably, in general, RNA-dependent mRNA amplification is, apparently, activated under the ISR conditions, which, presumably, supply, via transcriptional/translational reprogramming, the component(s) of the RdRp complex that are “missing” under regular conditions. In cases of erythroid differentiation, it appears that the ISR is elicited via the activation of the heme-regulated HRI kinase, which, in turn, is triggered by the deficiency of free heme; the deficiency arises due to the increased conventional production of globin and the resulting sequestration of heme within the hemoglobin tetramer structure. As in Alzheimer’s disease (discussed above), the activation of the RNA-dependent globin mRNA amplification and, eventually, greatly elevated production of globin polypeptides, creates the Engine that maintains the ISR and thus drives the amplification process: this occurs because as more globin is produced, more heme is sequestered, and its deficiency, and consequently the activity of HRI, are sustained. In the case of laminins, the increased conventional production of the ECM proteins apparently saturates the endoplasmic reticulum and causes the ER stress, thus activating PERK, another eIF2α kinase, and triggering the elicitation of the ISR and consequently of RNA-dependent mRNA amplification. The dramatically increased production of laminins (and other ECM proteins) sustains the ER stress, maintains the ISR conditions, and propagates the operation of the RNA-dependent mRNA amplification pathway [162].

In addition to the competent RdRp, the operation of the RNA-dependent mRNA amplification pathway requires the occurrence of an amplification-eligible RdRp mRNA template. In the examples presented above, both alpha and beta globin mRNA, as well as all three laminin mRNA species, are eligible RdRp templates [160,161,162]. The requirements for such eligibility, described in the preceding sections, are strict. Some requirements are inviolable, such as, for example, the lack of the 3′-terminal overhang within the TCE–ICE complex. But “loosening” of more minor requirements also significantly affects the efficiency of the amplification process. For example, even single-nucleotide changes within the 5′UTR of human beta globin mRNA, that do not affect the efficiency of its translation but reduce the extent of its TCE–ICE complementarity, interfere with its amplification and cause beta thalassemia [164]. It follows that since mice possess functional RNA-dependent mRNA amplification machinery, and since the ISR is elicited and RdRp complex is, presumably, assembled in the neurons of the current transgenic animal models of AD, the highly plausible deficiency preventing the operation of the AβPP-independent *i*Aβ production pathway is, as discussed in the following section, the lack of the RdRp-compatible, RNA-dependent amplification-eligible AβPP mRNA.

## 18. Mouse AβPP mRNA Is Ineligible for RNA-Dependent Amplification and So Are AβPP mRNAs Produced in the Current Models of AD from Human AβPP Transgenes

### 18.1. Mouse AβPP mRNA Is Not a Competent RdRp Template and Therefore Is Ineligible for RNA-Dependent Amplification

In humans, the AβPP-independent *i*Aβ production pathway could, apparently, be powered by the RNA-dependent amplification of AβPP mRNA. Activating this pathway is then synonymous with assembling the competent RdRp complex. In these terms, and according to the discussion in the preceding sections, the transcriptional/translational reprogramming by the integrated stress response provides missing component(s), potentially co-factors, of the RdRp complex. With the competent RdRp complex assembled, for the mRNA amplification process to occur, the only needed companion is an RdRp-compatible RNA template, i.e., an mRNA, which is eligible for the RdRp-mediated amplification by virtue of satisfying two requirements: 1. The occurrence of the TCE and ICE elements within the antisense RNA strand and 2. The topological compatibility of the TCE and ICE elements, i.e., their mutual accessibility in the folded antisense RNA configuration. As discussed above, human AβPP mRNA constitutes just such an RdRp-compatible, RNA-dependent amplification-eligible template.

**Figure 11:**
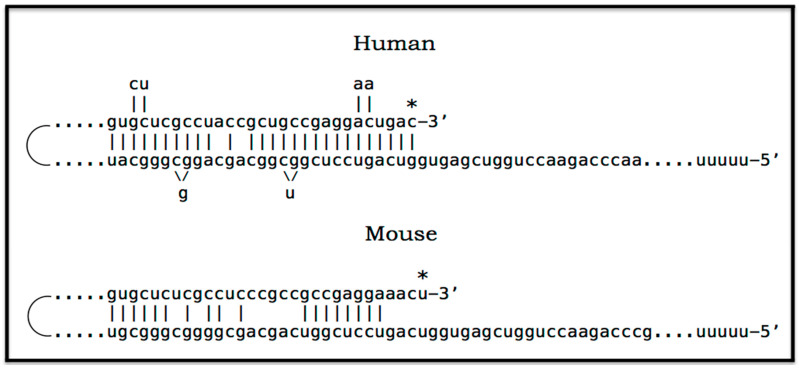
Mouse AβPP mRNA is ineligible for the amplification process: comparison of the relationships of the TCE and ICE elements within human antisense AβPP RNA and of analogous segments within mouse antisense AβPP RNA. *“Human”*: human antisense AβPP RNA folded into self-priming configuration. “Mouse”: the relationship between the analogous segments of the mouse antisense AβPP RNA. *Asterisk*: the nucleotide position on the antisense RNA corresponding to the TSS of human or mouse AβPP mRNA (in both cases, position (−)149 upstream from the “A” of the translation-initiating AUG codon). Note that the relationship between the 3′-terminal segment of the mouse antisense AβPP RNA and its internal segment corresponding to the TCE and ICE elements within human antisense AβPP RNA is not much stronger than random, and a substantial overhang at the 3′ terminus of the mouse RNA prohibits its utilization as a transcription primer.

Mouse AβPP mRNA, on the other hand, *is not* a legitimate RdRp template. For this mRNA species, the second requirement formulated above, that of an appropriate antisense RNA folding requirement, may or may not be satisfied. That this requirement is satisfied in human AβPP mRNA does not signify that it is also satisfied in its mouse counterpart; although the two encode nearly identical polypeptides, over one-third of nucleotides in these two RNA species are different, and the folding patterns of their antisense RNA counterparts could be highly diverse. This consideration, however, is irrelevant in the context of the present discussion. This is because in mouse AβPP mRNA, the first requirement, namely, the occurrence of the TCE and ICE elements within the antisense RNA strand, is clearly not satisfied. Upon the analysis of the nucleotide sequence of the mouse antisense AβPP RNA, it become apparent that its 3′-terminal segment does not have, within the entire AβPP antisense RNA, a sufficiently complementary companion that could allow the formation of a stable double-stranded structure with no 3′ overhang [173,174]. Indeed, as shown in Figure 11, in terms of complementarity, the relationship between the 3′-terminal segment of the mouse antisense AβPP RNA and its internal segment corresponding to the TCE and ICE elements within human antisense AβPP RNA is not much stronger than random, and a substantial overhang at the 3′ terminus of the mouse RNA prohibits its utilization as a transcription primer.

### 18.2. mRNA Products of Human AβPP Transgenes in the Current Animal AD Models Are Also Ineligible for the RNA-Dependent Amplification Process

If, upon the elicitation of the ISR in the current transgenic animal AD models (and, as discussed above, the ISR is elicited in these models via the accumulation of AβPP-derived *i*Aβ), the RNA-dependent mRNA amplification-competent RdRp complex is assembled (and, as discussed above, it is highly plausible that it is), it could be argued that since human AβPP mRNA is the eligible RdRp template, and because it is human AβPP mRNA, which is expressed from the transgenes in the current animal AD models, its amplification should occur. This, however, is not the case. The amplification depends on the highly delicate and precise TCE-ICE interaction, which occurs within the antisense complement of the *intact* human AβPP mRNA. But human AβPP mRNA expressed in the current transgenic animal AD models *is not intact*, i.e., not identical to endogenous human AβPP mRNA. The 5′-terminal portion of these transgenes, the very segment harboring the TCE element in the “intact” mRNA, is heavily modified during their construction. Consequently, the 5′UTR of the resulting mRNA is substantially changed and so is the 3′-terminal segment of the corresponding antisense AβPP RNA. Since this is the segment containing the TCE element, this element is profoundly altered and loses both its ability to interact with the ICE and its functionality as the transcription primer.

## 19. If Attempted, the “Legitimization” of Endogenous Mouse AβPP mRNA Transcripts via Facilitation of the TCE–ICE Interaction Would Likely Be Futile Due to the Thermodynamics of RNA Folding

Mouse Aβ differs in amino acid content from its human counterpart, and, in the past, attempts have been made to generate mouse models of AD by “humanizing” its Aβ. These attempts were unfruitful. In light of the ACH2.0 and of the considerations presented above, a different type of “humanization” of mouse AβPP gene could appear justified. Thus, it could be argued that if the endogenous mouse AβPP gene is modified so that its transcripts contain sufficiently complementary TCE and ICE elements lacking the 3′ overhang, the elicitation of the ISR via overexpression of exogenous Aβ or by other means (discussed above) would activate RNA-dependent amplification of the endogenous AβPP mRNA and consequently the AβPP-independent *i*Aβ production pathway, and that this, in turn, would trigger the commencement and progression of AD. This, however, is implausible because the second requirement for the functionality of the TCE–ICE elements (the first being a sufficient complementarity and the lack of the 3′-terminal overhang), namely, their mutual accessibility in the folded antisense RNA configuration, is unlikely to be satisfied.

Indeed, the change of only a few (possibly a single) nucleotides in key positions can radically alter the thermodynamics of the RNA folding process. In mouse AβPP mRNA, over a third of nucleotides differ from those in the corresponding positions in human AβPP mRNA (although their amino acid contents remain highly analogous); the same applies to their antisense RNA counterparts. It can be anticipated with a great degree of certainty that the folding conformation of mouse antisense AβPP RNA is substantially different from that of its human counterpart. Consequently, there is a significant probability that the TCE and ICE elements, “legitimized” by arranging their sufficient complementarity, would nevertheless be mutually inaccessible in the folded configuration of mouse antisense AβPP RNA.

## 20. Design of the Transgene Encoding Human AβPP mRNA Eligible for RNA-Dependent Amplification

### 20.1. Transcription of Human AβPP mRNA Can Be Initiated at the Multiple Locations; Only One of Them Is Compatible with the Amplification Process

**Figure 12:**
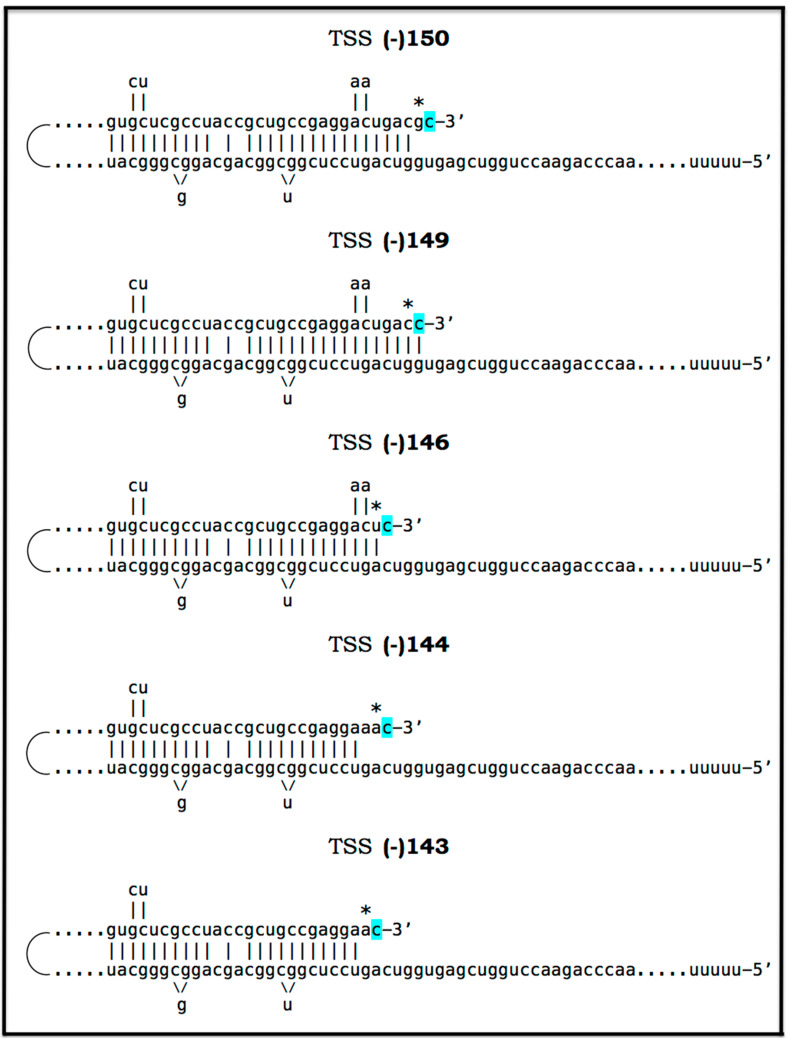
Folding of the antisense RNA originated from human AβPP mRNA initiated at the multiple TSS positions. *TSS*: transcription start site. (−)*150*, (−)*149*, (−)*146*, (−)*144*, (−)*143*: positions of the known TSSs of human AβPP mRNA. *Asterisk*: the nucleotide position on the antisense RNA corresponding to the TSS of human AβPP mRNA. Highlighted *in blue*: “C” transcribed from the 5′-terminal cap “G” of AβPP mRNA (not encoded in the genome). Shown in the figure are the interactions of the 3′-terminal segment of each antisense RNA type with its complementary counterpart within the molecule. Note that only one type of the antisense AβPP RNA species, namely, that generated from AβPP mRNA initiated at the TSS (−)149, would contain fully functional TCE and ICE elements capable of forming a self-priming structure. In the other four antisense AβPP RNA types, the 3′-terminal overhangs would preclude self-priming or at least substantially reduce its efficiency.

The AβPP gene belongs to the category of the TATA-less genes, i.e., genes lacking the “TATA box” as their control element. When present, the TATA box defines the precise position of the initiation of transcription, the “transcription start site,” TSS. In its absence, transcription usually initiates at multiple sites, albeit within narrow margins, i.e., within relatively small gene segment. This is indeed the case with the human AβPP gene. Within this gene, there are five known TSS sites [171]. They are situated at the following positions (distances in nucleotides counts are given from the “A” of the translation-initiating AUG codon): (−)150, (−)149, (−)146, (−)144, and (−)143 (corresponding positions on the antisense RNA are marked by asterisks in Figure 12). What is important for the present considerations is the requirement that there is no 3′-terminal overhang within the TCE–ICE complex in the folded antisense RNA. When analyzing the compliance with this requirement for AβPP antisense RNA generated from human AβPP transcripts initiated at each of the five TSS positions, one additional prerequisite should be taken into consideration: the additional 3′-terminal “C” transcribed by RdRp from the 5′-terminal cap “G”, not encoded in the genome, must be accommodated in the double-stranded TCE–ICE structure. This is because it was shown that RdRp is capable of transcribing the cap “G” of an mRNA template; this phenomenon was observed in all cases of RNA-dependent mRNA amplification analyzed to date, and thus appears to be the general case [160,161,162]. With the inclusion of the 3′-terminal genome-unencoded “C” transcribed by RdRp from the cap “G” of mRNA, the potential TCE–ICE complexes (“potential” because not all would be functional) within the corresponding antisense RNAs would appear as presented in Figure 12.

As shown in Figure 12, the analysis of the interaction of the 3′-terminal segment of the antisense RNA produced by RdRp from human AβPP mRNA initiated at the various TSS positions with its potential ICE counterpart indicates that only one type of the antisense AβPP RNA species, namely, that generated from AβPP mRNA initiated at the TSS (−)149, would contain fully functional TCE and ICE elements capable of forming a self-priming structure. In other four antisense AβPP RNA types, the 3′-terminal overhangs would preclude self-priming or at least substantially reduce its efficiency.

### 20.2. The Key to Success: Precisely Defined Initiation of Transcription from Human AβPP Transgenes Would Produce RdRp-Compatible AβPP mRNA Eligible for the RNA-Dependent Amplification Process

It follows from the preceding subsection that the key to success in generating a transgenic animal model capable of operating the AβPP-independent *i*Aβ production pathway and consequently of supporting the commencement and progression of AD is to utilize transgenes expressing human AβPP mRNA initiated at the TSS in position (−)149. One of the approaches to ensure the initiation of transcription from this particular position is to place human AβPP transgenes under the control of the appropriately positioned TATA box. In such experimental system, the elicitation of the ISR, either via the accumulation of exogenous *i*Aβ over the T1 threshold or by other means discussed above, would be followed by the transcriptional/translational reprogramming and the production of the “missing” component(s) required for the functional, amplification-competent RdRp complex. With the latter assembled, the presence of the RdRp-compatible, RNA-dependent amplification-eligible AβPP mRNA template would enable the operation of the amplification process and consequently of the AβPP-independent *i*Aβ production pathway. This in turn would empower the commencement and progression of AD.

## 21. “One-Stage” Construction of an Adequate AD Model: Analogues of the Current Transgenic Animal AD Models Expressing Amplification-Eligible Human AβPP mRNA Would Efficiently Emulate the Disease

The preceding sections addressed the principles of the “two-stage” construction of a transgenic animal model of AD. In such an approach, the origins or the nature of the agents eliciting the integrated stress response and those produced via the RNA-dependent amplification of human AβPP mRNA and driving the disease are different. In these models, the first stage, elicitation of the ISR, is enacted either by *i*Aβ produced from amplification-ineligible AβPP mRNA expressed by the exogenous transgenes (the current transgenic AD models are, in fact, the “first-stage” models), by *i*Aβ42 expressed from C100-encoding transgenes, or by various stressors other than *i*Aβ. The essence of the second stage is the RNA-dependent amplification of exogenous RdRp-compatible human AβPP mRNA and the resulting generation of *i*Aβ in quantities sufficient to drive the AD pathology (and to perpetuate its own production). Such models are “two-stage” because even if we utilize the current AD models as the “first-stage,” express *i*Aβ42, or elicit the ISR independently of *i*Aβ to enable the “second-stage,” we still have to introduce transgenes expressing human AβPP mRNA eligible for the amplification process. Would it not be simpler to do only the latter?

Indeed, the construction of a single-stage model can, with one exception, reproduce that of the current transgenic animal models of AD. The exception is that the transgenes should be designed and constructed in such a way as to express human RdRp-compatible and consequently RNA-amplification eligible AβPP mRNA. As discussed in the preceding sections, we know that the quantities of exogenous *i*Aβ expressed from human transgenes and derived conventionally in the AβPP proteolytic pathway would be sufficient to cause the activation of the eIF2α kinases and to trigger the elicitation of the ISR in neuronal cells. This would enable the assembly of the amplification-competent RdRp complex, and, provided exogenous AβPP mRNA is amplification-eligible, its RNA-dependent amplification would follow. This process would enable the operation of the AβPP-independent *i*Aβ production pathway, which in turn would empower the commencement and progression of AD.

Paradoxically, the single-stage model described above could be “too” efficient. In AD, RNA-dependent amplification of AβPP mRNA is based on the conventional expression of a single gene. In a human neuronal cell-based AD model, endogenous amplification of AβPP mRNA, transcribed from a single gene, presumably causes formation of NFTs in just a few days. In the proposed one-stage transgenic animal model of AD, the amplification of human AβPP mRNA would be based on the conventional exogenous expression of dozens, possibly around hundred, transgenes. This would increase the rate of *i*Aβ accumulation by an additional two orders of magnitude. As a result, the duration of the AD pathology could be significantly condensed, and the pathology itself possibly altered; this could make it difficult to be used in some applications, such as the testing of potential AD drugs. In such a case, the two-stage approach should be employed in the construction of a model: the number of transgenes expressing RdRp-compatible amplification-eligible human AβPP mRNA should be minimized, and the ISR should be elicited via the expression of amplification-ineligible AβPP or Aβ mRNA or through utilization of stressors other than *i*Aβ.

Alternatively, a current transgenic animal model of AD can be manipulated with a similar effect. Human AβPP transgenes in such a model could potentially be edited with the double aim of (a) restoring the 5′ termini of transgenes to their origin and (b) ascertaining that their transcription would initiate at the TSS (−)149. Accomplishing this would lead to the production of RdRp-compatible RNA-dependent amplification-eligible human AβPP mRNA transcripts. If only a fraction of the AβPP transgenes in a current transgenic AD model were successfully edited along the above lines, it could be sufficient to generate an adequate animal model of the disease.

## 22. Conclusions

The present study examines the suitability of the current transgenic models of Alzheimer’s disease. It concludes that these models are inadequate, and, moreover, that their inadequacy reflects that of the theory of AD that guided their design—the ACH. Furthermore, the insufficiencies of the ACH and of ACH-based AD models also encompass the inefficiency of ACH-based AD drugs, emphasizing the inextricable connection between the three. These shortcomings have necessitated the formulation of a new theory of AD that is consistent with the accumulated empirical data, fully explains the phenomenology of the disease, and can serve as a basis for the design and construction of adequate AD models and effective AD drugs.

The recently introduced novel interpretation of AD, referred to as ACH2.0 since it retains the centrality and the causative role of Aβ in the disease, appears to constitute just such a theory. It posits that AD is driven by *i*Aβ produced independently of AβPP and retained intraneuronally. The AβPP-independent *i*Aβ production pathway in neuronal cells is activated as a consequence of the integrated stress response. The ISR initiates transcriptional/translational reprogramming, and some of the newly synthesized proteins provide the necessary component(s) of the AβPP-independent *i*Aβ generation pathway, which are missing under normal conditions, and thus enable its operation. The ISR in neuronal cells is elicited via the phosphorylation of eIF2α at its serine residue 51. This is enacted by one (or more) of the four eIF2α kinases PERK (PKR-like ER kinase), PKR (protein kinase double-stranded RNA-dependent), GCN2 (general control non-derepressible-2), and HRI (heme-regulated inhibitor). In conventional AD, AβPP-derived *i*Aβ, accumulated over the critical threshold, triggers the neuronal activation of the PKR and/or HRI kinases, whereas in unconventional AD, one or more of the eIF2α kinases are activated in neurons by stressors other than *i*Aβ. The levels of *i*Aβ produced in the AβPP proteolytic pathway alone are insufficient to cause and support the progression of AD; the disease commences only following the activation of the AβPP-independent pathway of *i*Aβ generation. Within the framework of the disease, *i*Aβ produced independently of AβPP performs two principal functions: it drives the AD pathology and sustains the ISR condition, thus propagating its own production and perpetuating the operation of the AβPP-independent *i*Aβ production pathway, a cyclical process referred to as the AD Engine.

Apparently, the principal problem with the current transgenic animal models of AD is that, for the reasons elaborated in the preceding sections, they lack the operational AβPP-independent *i*Aβ production pathway. They model, in fact, not Alzheimer’s disease but rather the effects of the neuronal ISR sustained by AβPP-derived *i*Aβ. This explains why they are incapable of developing the full spectrum of AD pathology, and why ACH-based drugs were spectacularly successful in current transgenic AD models, but completely inefficient in treating the disease. The present study concludes that any adequate transgenic animal model of AD must incorporate the operational AβPP-independent pathway of *i*Aβ generation. It also discusses cellular mechanisms potentially enabling the operation of the AβPP-independent *i*Aβ generation pathway, posits the principles of design of adequate, physiologically suitable ACH2.0-guided models of AD, and describes the molecular details of their construction.
